# Influence of Diluent on Extraction Parameters of Systems for Separation Am(III) and Ln(III) Based on 1,10-Phenanthroline-2,9-Diamide

**DOI:** 10.3390/molecules29153548

**Published:** 2024-07-28

**Authors:** Mikhail A. Gerasimov, Petr I. Matveev, Mariia V. Evsiunina, Enni. K. Khult, Paulina Kalle, Valentine S. Petrov, Pavel S. Lemport, Vladimir G. Petrov, Galina V. Kostikova, Yuri A. Ustynyuk, Valentine G. Nenajdenko

**Affiliations:** 1 Department of Chemistry, Lomonosov Moscow State University, Moscow 119991, Russia; mishasmt@mail.ru (M.A.G.); petr.i.matveev@gmail.com (P.I.M.); mashko-ya-e@mail.ru (M.V.E.); vs.petrov25@gmail.com (V.S.P.); vladimir.g.petrov@gmail.com (V.G.P.); yuriustynyuk@gmail.com (Y.A.U.); 2 Department of Materials Science, Lomonosov Moscow State University, Moscow 119991, Russia; 3 N.S. Kurnakov Institute of General and Inorganic Chemistry of the Russian Academy of Sciences, Moscow 119991, Russia; kalle@igic.ras.ru; 4 Frumkin Institute of Physical Chemistry and Electrochemistry of the Russian Academy of Sciences, Moscow 119071, Russia; galyna_k@mail.ru

**Keywords:** *f*-element, liquid extraction, solvent, ionic liquid, mechanism of complexation

## Abstract

A systematic study of extraction systems for the separation of *f*-elements using the tetradentate N,O-donor diamide of 1,10-phenanthroline-2,9-dicarboxylic acid (**L**) in various molecular and ionic solvents was performed. It was demonstrated that the nature of a diluent has a significant impact on solvent extraction of Am(III) and Ln(III) and the stoichiometry of formed complexes with *f*-elements. The mechanism of complexation and forms of complexes in different diluents were investigated by radiometric methods, UV-vis titration, and XRD.

## 1. Introduction

The main challenge in nuclear energy production lies in managing high-level radioactive waste (HLW) generated during the reprocessing of spent nuclear fuel (SNF) [1]. The only method currently used for the industrial recycling of SNF is the PUREX process (Plutonium Uranium Reduction EXtraction) [2,3,4]. After the extraction of uranium and plutonium, the residual radiotoxicity is primarily associated with minor actinides (MA), especially ^241^Am (T_1/2_ = 432.6 years), ^243^Am (T_1/2_ = 7370 years), and ^244^Cm (T_1/2_ = 18.1 years). A progressive approach to addressing this issue is the concept of partitioning and transmutation [1,3]. Recent initiatives focus on exclusively transmutating americium to diminish long-term radiotoxicity and optimize the storage space in geological repositories. Developing a solvent extraction process is pivotal for recovering this *f*-element from PUREX raffinate [5].

Two main challenges involve separating americium from fission products (mainly lanthanides, which act as neutron poisons) and eliminating curium (a powerful source of neutrons, limiting its use due to the need for extensive shielding at any stage of the nuclear fuel cycle) [6]. Several processes, including PUREX-DIAMEX-LUCA [7] and EXAm [2], have been proposed. However, despite ongoing efforts, a reprocessing system that meets all technological criteria remains elusive.

Developing a technological scheme for implementing the partitioning and transmutation concept for minor actinides involves several key stages, including selecting an extractant and organic solvent, as well as conducting static and dynamic tests [6,8]. When selecting components for the extraction system for effective Am(III) recovery, the following parameters must be considered: the values of the distribution ratios of the extracted component (D(Am)) should range from 1 to 10 since D < 1 implies that a large fraction of the component will remain in the aqueous phase, and significantly high values of D (>1000) make back-extraction processes difficult or impossible. The minimum value of the separation factor between two components allowing industrial separation is estimated as SF (Am/component HLW) ≥ 5 [3]. The larger the separation factor, the fewer stages will be required to achieve the needed product purity.

Diamides of 1,10-phenanthroline-2,9-dicarboxylic acids (DAPhen) are promising extractants. Due to the combination of the soft nitrogen atoms of the phenanthroline fragment of the extractant with the hard oxygen atoms of the amide groups, this class of extractants demonstrates good selectivity for Am(III) over Ln(III) and Am(III) over Cm(III) [6,8,9,10,11,12,13,14,15,16,17,18,19,20]. The results obtained for these ligands show the prospect of their industrial-scale use, especially from nitric acid solutions, as these extractants exhibit a rapidly establishing extraction equilibrium, which is important for modeling dynamic tests [9].

The proper selection of the solvent in modeling the extraction system is an essential task. This is the basis of the technological scheme for partitioning HLW since it constitutes a significant portion of the organic phase. Accordingly, all safety-related requirements mainly pertain to the properties of these compounds. Also, the price of the diluent is an important parameter [15,16,21,22].

**Figure 1:**
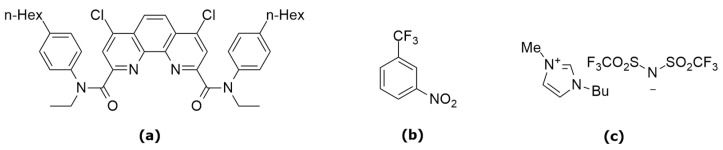
Structure of N,N’-diethyl-N,N’-di(4-hexyl-phenyl)-diamide of 1,10-phenanthroline-4,7-dichloro-2,9-dicarboxylic acid—**L** (**a**); *m*-nitrobenzotrifluoride—**F-3** (**b**) and 1-butyl-3-methylimidazolium bis(trifluoromethanesulfonyl)imide—**C_4_mimNTf_2_ (IL)** (**c**).

Hydrocarbons are most commonly used as solvents for extractants in radiochemical practice. However, the solubility of polar ligands, such as derivatives of 1,10-phenanthroline-2,9-dicarboxylic acids, is limited in hydrocarbons. In this case, the possible formation of third phases can be expected, which is undesirable for technological applications [4]. More often, polar solvents are used as diluents for extraction systems based on extractants of this type. The most promising results have been obtained using for this aim ionic liquids [22,23,24,25] or fluorinated solvents like *m*-nitrobenzotrifluoride (F-3) (Figure 1) [4,6,8].

When using various DAPhen derivatives in combination with the ionic liquid C_4_mimNTf_2_ as a solvent, the cation-exchange mechanism prevails in the system, leading to the formation of a 1:2 (metal:ligand) complex in the organic phase. This explains the significant enhancement of the ligand’s extraction ability in ionic liquids compared to conventional molecular solvents [23,24]. However, these systems have various disadvantages, including stability only in weakly acidic solutions, a prolonged establishment of the extraction equilibrium, high solubility in water (especially in the presence of nitric acid), and high viscosity. Furthermore, this type of diluent may pose greater risks to human health compared to molecular solvents, raising concerns about the designation of ionic liquids as “green” solvents [26].

It is worth noting that the extraction ability of a ligand in different solvents can vary significantly due to different complexation mechanisms. This behavior may correlate with solvent properties such as the dielectric constant, molecular weight, or viscosity. Some research findings suggest that extraction systems employing nonpolar organic diluents exhibit a lower separation factor SF(Am/Eu) compared to more polar solvents. Additionally, solvents with lower dielectric constants may demonstrate increased efficiency at higher acid concentrations due to their neutrality. These results raise questions about potential synergistic effects and competition with HNO_3_ as well as neutral DAPhen ligands. Also noteworthy is the lack of systematic studies of various solvents for extraction systems based on N,O-donor ligands [16,20].

We decided to study the extraction ability of N,N’-diethyl-N,N’-di(para-hexyl-phenyl)-diamide of 1,10-phenanthroline-4,7-dichloro-2,9-dicarboxylic acid (L) (Figure 1) as an extractant for the separation of MA/Ln in different molecular and ionic diluents. The choice of the ligand structure is determined by several factors, including a “strong” phenanthroline framework and the presence of halogens and aromatic fragments (the ability for π-π stacking and the formation of halogen bonds with solvent molecules).

The dependence of the physical properties of the solvent (dielectric constant, viscosity, polarizability) on the parameters of the extractant was investigated. Particular attention was paid to the structure of the complexes of *f*-elements in the studied systems depending on the solvent nature.

## 2. Results and Discussion

### 2.1. Solvent Extraction of Actinides (An(III)) and Lanthanides (Ln(III))

Solvent extraction is the most suitable method for separating and concentrating *f*-elements from HLW. The essence of this method lies in the distribution of components between two unmixable phases when they are in contact. The PUREX-process raffinate is a nitric acid solution containing a large number of components, including actinides(III) and lanthanides(III). The organic phase mostly consists of an extractant in a hydrophobic organic diluent. Since the solvent constitutes the majority of the organic phase, it can influence the complexation mechanism and, hence, the characteristics of the extraction systems. To investigate the influence of the solvent on the extraction parameters of systems based on N,O-donor ligand **L**, we considered molecular and ionic diluents with varying physicochemical properties (dielectric constant and viscosity). We studied a series of diluents with a wide dielectric constant range—from 2 to 35. The properties of diluents and approximate solubility of **L** in the solvents [8,27,28,29,30,31,32,33] are presented in Table S1. These solvents can be grouped according to their structure as follows:

- Solvents containing aromatic fragments in the structure (π-π stacking with ligand molecules is possible)—toluene, *m*-nitrobenzotrifluoride (F-3), and nitrobenzene;
- Chlorine-containing organic compounds (halogen–halogen interactions between ligand and solvent molecules due to overlapping p-orbitals of chlorine atoms)—chloroform and 1,2-dichloroethane;
- Aliphatic alcohols (hydrogen bonds)—octanol-1 and dodecanol-1;
- Ionic solvent—1-butyl-3-methylimidazolium bis(trifluoromethanesulfonyl)imide (C_4_mimNTf_2_).

In terms of further processing of used solvents, the CHON principle corresponds to aliphatic alcohols, toluene, and nitrobenzene.

It is worth noting that all the solvents studied in this work have different viscosities. This parameter is important as it affects the kinetics of the complexation process. Viscosity must also be taken into account when choosing a solvent. However, this parameter does not significantly affect the thermodynamics of the process of binding *f*-elements by the ligand L under study.

#### 2.1.1. Solvent Extraction of Am(III) and Eu(III)

**Figure 2:**
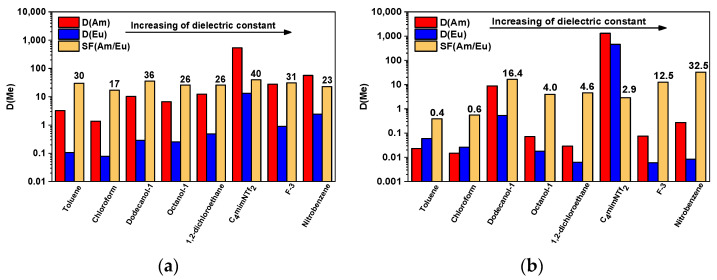
D(Am), D(Eu), and SF(Am/Eu) (**a**) for extraction and (**b**) back-extraction processes in different molecular diluents. Aqueous phase: 3 mol/L HNO_3_. Organic phase: 0.025 mol/L of **L**. t = 15 min. (for ionic liquid t = 1 h), T = 298 ± 1 K (*p* = 0.95; *n* = 3; SD < 10%).

The extraction of Am(III) and Eu(III) with 0.025 mol/L solutions of ligand L in various solvents from 3 mol/L HNO_3_ was studied. The dielectric constant consistently emerges as the most critical parameter for comparing extraction parameters across different diluents. [16,20]. In addition, it is presumed that the stability of complex compounds in the solvent directly correlates with the phase stability of the organic part of the system. This parameter should be directly related to the molarity of the diluent, that is, with the concentration of solvent molecules in itself. As can be seen from the data presented in Figure 2a (Table S2), there is a certain pattern—in most cases, the dielectric constant of the solvent and the distribution ratio increases in a symbatic way. For instance, although 1,2-dichloroethane and octanol-1 share similar dielectric constants, their observed distribution ratios differ significantly. Hence, the values of distribution ratios depend not only on the dielectric constant of the solvent but also on other factors.

The separation factors have the highest values in the case of ionic liquid, dodecanol-1, and toluene. These observations may be associated with specific interaction between **L** and the solvent or the influence of other diluent properties. It is interesting to consider the relationship between the SF(Am/Eu) and the dielectric constant of the molecular diluent.

The extraction system with a combination of ligand L and ionic liquid C_4_mimNTf_2_ demonstrated the most efficient separation. The values of distribution ratios at an **L** concentration of 0.025 mol/L were 537 and 13.4 for Am(III) and Eu(III), respectively, and SF(Am/Eu) = 40. The main problem of this system is low stability to form a third phase and a long time to establish extraction equilibrium. When the concentration of **L** in the organic phase exceeded 0.03 mol/L, a white precipitate fell out. Contacting the solution with the precipitate and nitric acid solution did not change the situation. It is important to note that the volume of the precipitate did not depend on the amount of dissolved ligand. We assumed that this is probably related to the chemical interaction between the extractant and the ionic liquid. However, on the HRMS ESI spectrum of the precipitate obtained in extraction systems, no interaction adducts were found—the mass spectrum contained only signals of the cation of the ionic liquid and **L** in the mass spectrum of positive ions and the anion of the ionic liquid in the case of negative ions (Figures S10–S12).

The ability of the extraction system, namely, the combination of ligand and solvent, to back-extraction is an important parameter in modeling the separation technology in terms of solvent recycling. In Figure 2b (Table S2), it can be observed that in the case of most molecular solvents, the distribution ratios of Am(III) and Eu(III) are less than 0.1, indicating almost complete transfer of these elements to the aqueous phase (0.5 mol/L HNO_3_). However, in the case of dodecanol-1, the back-extraction is partial—D(Am) = 8.8 and D(Eu) = 0.54.

In the case of ionic liquids, there is no back-extraction. This is probably due to the stabilization of the complex in the organic phase. The combination of ligand **L** with an ionic liquid strongly binds *f*-elements in the organic phase. An alternative to back-extraction is evaporation/solvent stripping, which is also not realizable for this type of organic compound. This is due to their melting at higher temperatures. However, they can be used in a mixture with molecular solvents as synergistic additives [34].

To establish a pattern between SF(Am/Eu) and the solvent used, additional parameters must be considered. The dielectric constant alone does not explain the observed changes in distribution ratios. For instance, in the case of 1,2-dichloroethane and octanol-1, their dielectric constants are similar, yet their polarizabilities differ. Perhaps polarizability could serve as an additional criterion when selecting a solvent based on the extraction properties of the ligand.

#### 2.1.2. Solvent Extraction of Ln(III)

**Figure 3:**
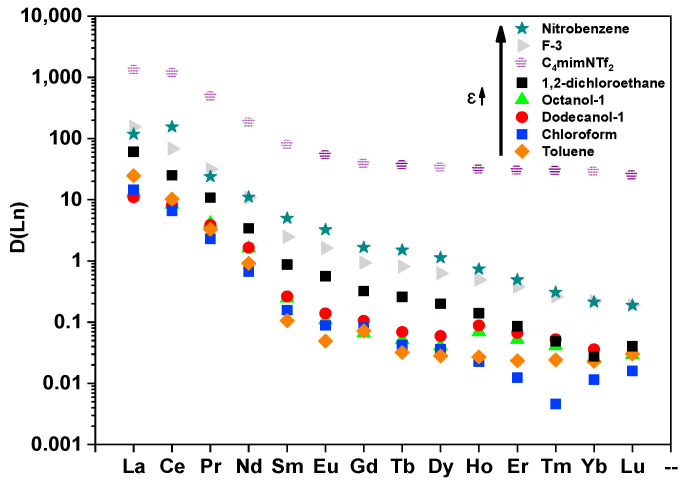
D(Ln) in different diluents. Aqueous phase: 3 mol/L HNO_3_. Organic phase: 0.025 mol/L of L. t = 15 min. (for ionic liquid t = 1 h), T = 298 ± 1 K (*p* = 0.95; *n* = 10; number of scans = 10 SD < 2%).

The extraction of all Ln(III) (except Pm) from a 3 mol/L nitric acid solution was studied. Ligand **L** (C(L) = 0.025 mol/L) was used as the extractant in various solvents. The same trend is observed in all cases: the distribution ratios decrease from La to Lu (Figure 3, Table S3). It can also be noted that in most cases, the distribution ratio for each lanthanide increases with the increase in the dielectric constant of the solvent.

In the case of ionic liquid, the distribution ratios of Ln(III) are higher than in all molecular solvents. The general trend remains the same (the minimum in the area of Tb and Dy). However, there is a rise from Er with a maximum of Yb. However, in the case of extraction with molecular solvents, the general trend is a decrease in D with an increase in the atomic number of the lanthanide.

**Figure 4:**
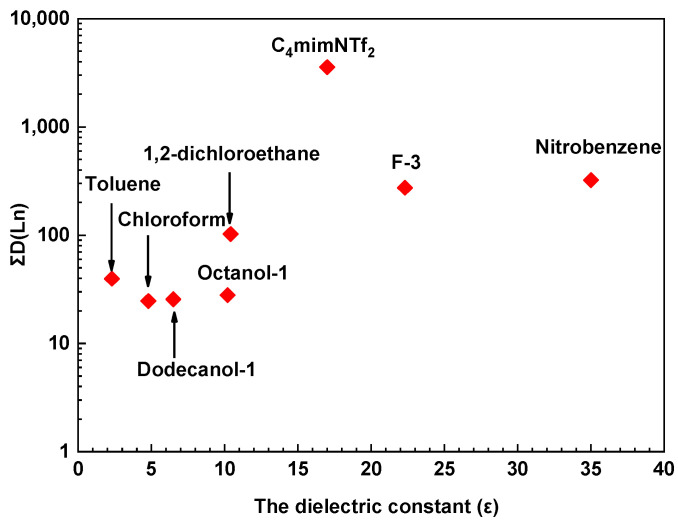
∑D(Ln) dependence on containing the organic phase for molecular diluents. Aqueous phase: 3 mol/L HNO_3_; organic phase: 0.05 mol/L of **L** in different diluents. T = 298 ± 1 K.

The dependence of the ∑D(Ln) (the sum of the distribution ratios of elements from La to Lu without Pm) obtained for molecular solvents on the dielectric constant also indicates that in a more polar solvent, the distribution ratio is higher than in a less polar one (Figure 4). However, in the case of 1,2-dichloroethane and 1-octanol, the values differ significantly, which underlines that the dielectric constant alone is not enough for solvent selection when modeling an extraction system. It should also be noted that ∑D(Ln) is significantly higher in the case of ionic liquid than in the case of any of the considered molecular solvents (∑D(Ln) > 3500).

Given the extraction parameters obtained for different model systems in all the solvents studied, it is interesting to investigate the processes leading to such different properties of the same extractant in these diluents. The likely influence of the solvent on the extraction properties is the possibility of forming aggregates of **L** in the organic phase. It may be connected with the molarity, polarizability of diluents, and the ability of hydrogen bonding/specific interaction between the **L** and solvent molecules [21,32,33,34]. The aggregation of ligands in organic phases has also been noted for diglycolic acid diamides [35,36]. This ligand forms reverse micelles in hydrocarbon diluents, with water in the core and around 4 molecules of extractant. It should be noted that the solvation numbers for TODGA (N,N,N,N Tetraoctyl Diglycolamide) in hydrocarbon solvents coincide with the amount of extractant in the micelle—thus, such a micelle, in a way, preorganizes the necessary amount of extractant molecules, which leads to an increase in extraction. On the other hand, the aggregation of micelles ultimately leads to the formation of a third phase. The same is true for extraction systems based on malonamides [37]. Thus, it can be stated that the observed difference in selectivity and extraction ability may be associated with specific states of the extractant in the solvent. Also, the solvent can form supramolecular complexes with ligand molecules or change its conformation, which will affect the parameters of extraction systems. Therefore, it is especially important to study the mechanism of complex formation between **L** and *f*-element in each solvent considered.

### 2.2. Complexation Study

#### 2.2.1. Solvation Numbers for Am(III) and Eu(III)

To determine the composition of the extracted complexes, the solvation numbers (SN) of Am(III) and Eu(III) were determined using the slope analysis (description and equations in Table S4). The solution of 3 mol/L HNO_3_ was used as the aqueous phase, and the ligand **L** dissolved in organic diluents was used as the organic phase.

Non-integer solvation numbers can be explained by the formation of several types of complexes in the organic phase. As can be seen in Table 1, the solvation numbers of Eu(III) for **L** in all molecular solvents are in the range of 1–1.4. That means that ML(NO_3_)_3_ and ML_2_(NO_3_)_3_ complexes can exist in the organic phase, with the complex with a metal:ligand ratio of 1:1 predominating. Solvation numbers for Am(III) in various solvents are in the range from 1.3 to 2.0. These data may indicate the formation of a mixture of 1:1 and 1:2 complexes, which are in equilibrium. However, in the case of toluene and chloroform, there is a clear predominance of the 1:2 complex for americium. A similar picture was observed for ionic liquid; complexes of composition 1:2 (M:L) predominate for both americium and europium according to experimental solvation numbers. Previously, we have shown that this phenomenon is related to the participation of NTf_2_^−^ in complexation between the ligand and cation [34].

#### 2.2.2. Saturation of Organic Phases

**Figure 5:**
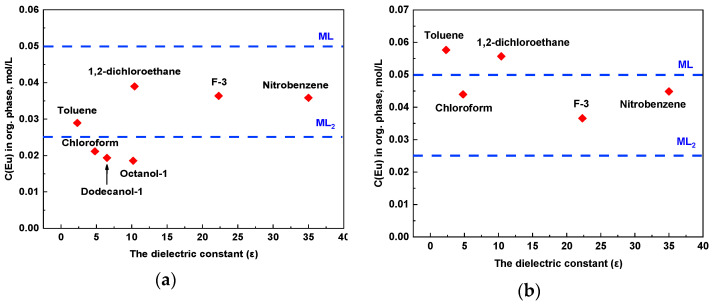
Saturation of organic phases. Aqueous phase: Eu^3+^ solution in 3 mol/L HNO_3_; (**a**) C(Eu^3+^) = 0.1 mol/L; (**b**) C(Eu^3+^) = 0.5 mol/L. Organic phase: 0.05 mol/L of **L**; T = 298 ± 1 K.

Next, we decided to determine the stoichiometry of complexes as well as to study the capacity of extractants using saturation of organic phases by Eu(III) (Figure 5). Solutions of 0.1 mol/L and 0.5 mol/L of stable Eu^3+^ with the addition of ^152^Eu^3+^ in 3 mol/L HNO_3_ were used as the aqueous phase.

According to the obtained dependencies, the following features can be highlighted for molecular solvents:

- Aliphatic alcohols—in extraction systems with C(Eu^3+^) = 0.5 mol/L, a precipitate fell out. Therefore, the system has low phase stability. The stoichiometry of the complex in the precipitate was calculated as the difference between the count rate of the initial solution and the sum of the count rates for the aqueous and organic phases, and it is equal to 1:1 (metal:ligand). The reason may be in the presence of specific interaction (hydrogen bonding) between complexes and alcohol molecules;
- Aromatic solvents F-3 and nitrobenzene—the concentration of Eu^3+^ practically does not change when the saturation occurs. Therefore, both 1:1 and 1:2 complexes are formed during saturation in these solvents;
- In the case of less polar toluene, chloroform, and 1,2-dichloroethane, the concentration values double when moving to a more saturated europium solution. This indicates a change in the stoichiometry of the complex in a saturated solution.

As a result, in the case of solvents with low dielectric constant values, 1:1 complexes are predominantly formed in the presence of a large excess of Eu(NO_3_)_3_. In the case of solvents with high polarity, the formation of 1:2 (metal:ligand) complexes is more favorable.

Next, the same experiments were performed with ionic liquid (C(L) = 0.025 mol/L). This system demonstrated a low phase stability to form precipitate via contact with 0.1 mol/L and 0.5 mol/L solutions of Eu(NO_3_)_3_. According to the data obtained with γ-spectroscopy, it was shown that radioactive Eu^3+^ is contained in the precipitate. Therefore, the formed precipitate is a europium complex with **L**. The count rate of the precipitate was calculated from the difference in the count rates of the original label and the sum of the aqueous and organic phases. This count rate was recalculated into the concentration of Eu^3+^ in the formed precipitate. For systems with an initial europium concentration of 0.1 mol/L and 0.5 mol/L in the aqueous phase, the content in the precipitate was 0.015 ± 0.001 mol/L and 0.031 ± 0.002 mol/L, and the L:M ratio in the obtained systems was 1.6 and 0.8, respectively. Therefore, the stoichiometry of the complex can be changed in the presence of a large excess of *f*-element in the system containing ionic liquid. A predominantly 1:1 complex is formed in this case.

#### 2.2.3. Spectrophotometric Titration

**Figure 6:**
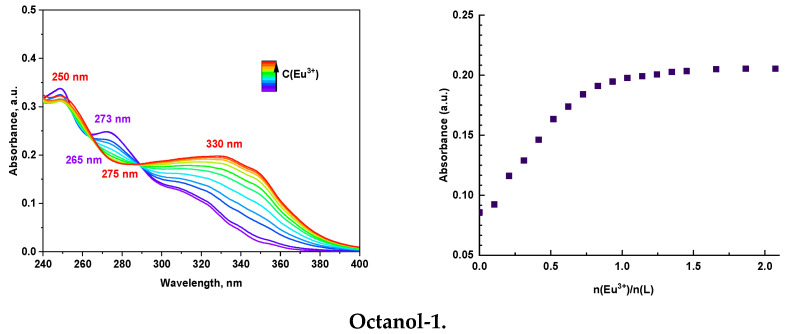

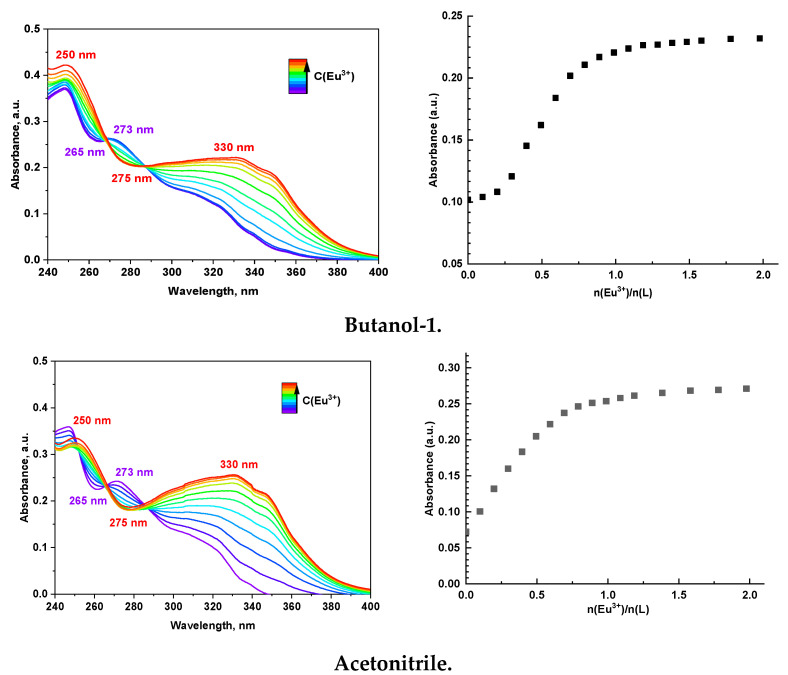
Absorption spectra and titration curves (λ = 330 nm) for **L** (C(L) ≈ 10^−5^ mol/L).

In order to establish the stoichiometry of the europium complex with **L** and to determine their stability constants, spectrophotometric titration was carried out. Isosbestic points are clearly visible on all absorption spectra (Figure 6). Therefore, a free ligand and a complex of this ligand with Eu(III) are present in the solution. The absorption maxima of the free ligand are observed at a wavelength of ~273 nm. The absorption maximum for EuL_n_ complexes ~330 nm coincides with UV-vis titration in acetonitrile, octanol-1, and butanol-1. Also, for these solvents, the absorption minimum of ~ 275 nm coincides, which indicates the same stoichiometry of the formed complexes. In all cases, complexes of composition 1:1 and 1:2 (M:L) are formed, and this is confirmed by titration curves (Figure 6). The most stable complexes are formed in acetonitrile, the least stable in octanol-1. The stability constants of the Eu(III) complex with L, obtained using the HypSpec2014 program [38], are presented in Table 2.

One can see that the solvent influences the stability constants. There is a correlation that with the increase in dielectric permittivity, the binding constant values increase. Therefore, more stable complexes are formed in solvents of higher polarity. Most probably, highly polar complexes ML and ML_2_ are preferably formed in solvents of higher polarity.

### 2.3. Approximate Solubility of **L** and MLn(NO_3_)_3_ Complexes in Different Diluents

The solubility of the extractant in a molecular solvent is an important parameter determining the effectiveness of the extraction system since it affects such an important parameter as capacity. This is because the concentration of f-element in the organic phase is connected with the L concentration due to lipophilic complex formation. In addition, a larger maximum solubility of the ligand in the diluent makes it easier to select optimal conditions for separation metals. For example, it allows for avoiding or reducing correction for the composition of the original aqueous solution containing the separated components. In this regard, the next step in studying the reason for the change in extraction capabilities of L when changing the solvent was to evaluate solubility.

According to Table S1, extractant **L** has higher solubility in 1,2-dichloroethane and chloroform. These solvents have the highest values of molarity (the concentration of solvent molecules in itself). The lowest solubility value is for octanol-1 and dodecanol-1, which also correlates with the values of molarity of the solvents. Therefore, the concentration of the solvent in itself and the maximum possible concentration of **L** in this solvent change symbiotically. Thus, for the development of an extraction system, it is necessary to consider solvents with a high value of molarity and low molecular weight.

The subsequent step of our study involved examination of the solubility of complexes of **L** with some *f*-elements. For these purposes, we synthesized complexes ML(NO_3_)_3_, where M = La, Eu, Nd, Lu. The structure of these complexes was confirmed by X-ray diffraction analysis (Section 2.4). In solubility tests, we found that the concentrations of saturated solutions in all these complexes were approximately 0.2 M in chloroform, 1,2-dichloroethane, and nitrobenzene. These three solvents have high molarity values, which seemed to be the most effective. However, the fact that we did not observe the same value in toluene, despite its high molarity, suggests that other factors (polarity) should be taken into account.

In the case of toluene, dodecanol-1, octanol-1, F-3, and ionic liquid, when the solvent was added portion-wise, the sample of complex (m = 5 mg) did not dissolve, and after adding 200 µL of solvent, a precipitate fell out, which did not dissolve with further addition of solvent. Probably, this is due to an additional competing process—the dissociation of the complex into components. In connection with this assumption, 3 mol/L nitric acid (50 µL) was added to the samples containing the third phase. In the case of theses complexes of lanthanides, nitrate anion enters the inner coordination sphere, and the presence of H_3_O^+^ and H_2_O in the solution the stability of complexes increased. The addition of acid affected the system with the solvent F-3—the precipitate dissolved. However, in other cases, the third phase remained unchanged.

### 2.4. X-ray Diffraction Method

**Figure 7:**
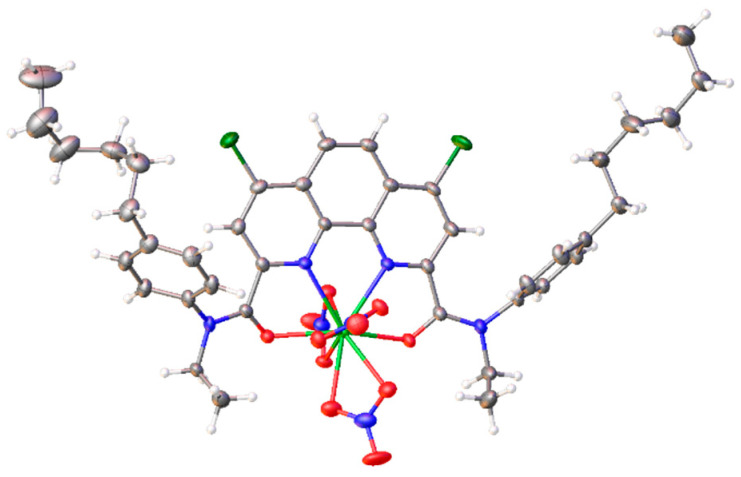
Molecular structure of LuL(NO_3_)_3_ (isostructural with NdL(NO_3_)_3_). Displacement ellipsoids are shown at the 50% probability level.

**Figure 8:**
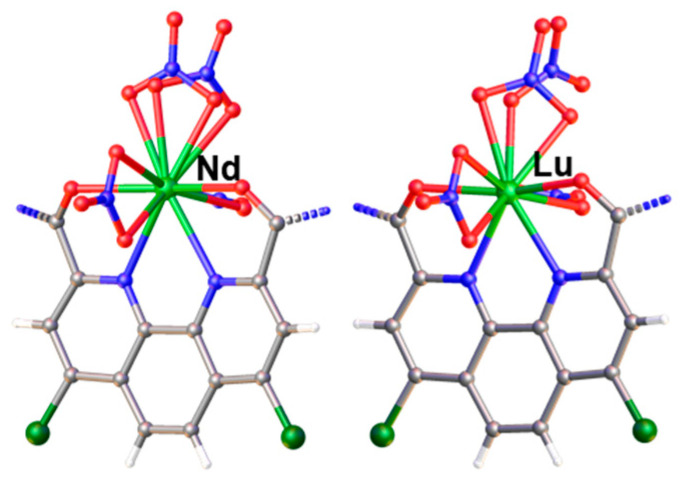
Disordered nitrate group in complexes NdL(NO_3_)_3_ and LuL(NO_3_)_3_. The central fragment of the complex is shown.

Single crystals of NdL(NO_3_)_3_ (CCDC number 2361229) and LuL(NO_3_)_3_ (CCDC number 2361230) were grown from a chloroform/acetonitrile/toluene mixture. However, the crystal structures do not include any solvent molecules. Both complexes crystallize in the non-centrosymmetric space group Pca2_1_ and are isostructural to the europium complex with the same ligand **L** [13]. There are two independent neutral molecules with similar structures in the asymmetric unit. The metal cation is coordinated by the tetradentate ligand and by three nitrate counterions (Figure 7). In the lutetium complex, the NO_3_^−^ group opposite to the phenanthroline moiety is asymmetrically coordinated, with one Lu−O bond 2.399(5) Å and the other 2.515(5) Å, while the rest of the Lu−O_nitrate_ distances are in the range of 2.423(4)−2.455(4) Å. In the second independent complex of lutetium, this nitrate group is disordered over two positions, with the main component (75%) being in the monodentate mode. The neodymium complex, as well as the published europium complex, do not possess any asymmetry in the nitrate binding. The disorder of the nitrate group also has a different nature and includes only bidentate nitrate groups (Figure 8). Differences in the structures are caused by steric strains appearing with the decrease in the ionic radius of the lanthanide.

**Figure 9:**
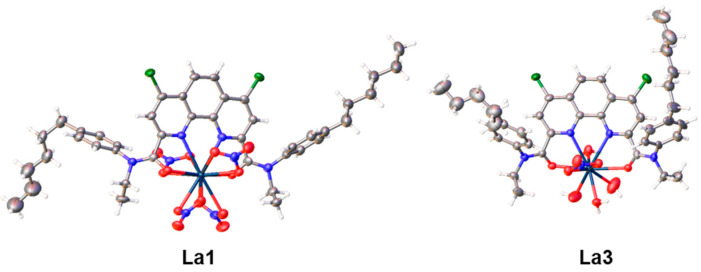
Molecular structures of lanthanum complexes with L. Displacement ellipsoids are shown at the 50% probability level.

The addition of ethanol to the crystallization solvent mixture had an impact on the crystal structure of the lanthanum complex with **L** (CCDC number 2361228). The triclinic (*p* – 1) structure contains free water and ethanol molecules. In the asymmetric unit, there are three independent complexes forming, in total, a neutral agglomerate. Two complexes (La1, La2) are anionic and have the composition [LaL(NO_3_)_4_]^−^, while the third (La3) complex is cationic and includes coordinated water molecules [LaL(NO_3_)(H_2_O)_4_]^2+^ (Figure 9). The two 12-coordinated lanthanum ions are in a distorted icosahedral environment. The geometry of the third lanthanum ion is difficult to assign to any regular polyhedron.

**Figure 10:**
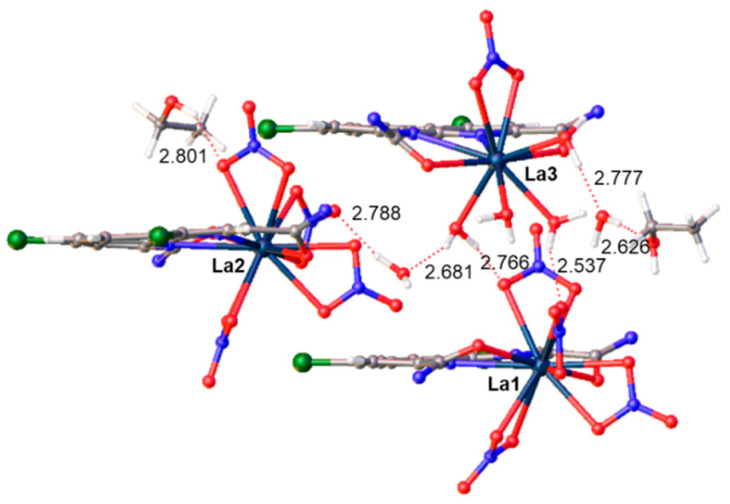
Hydrogen bonds in the asymmetric unit of complex La(NO_3_)_3_ with **L**. Donor–acceptor distances are given in Å. The amide substituents are omitted for clarity.

**Figure 11:**
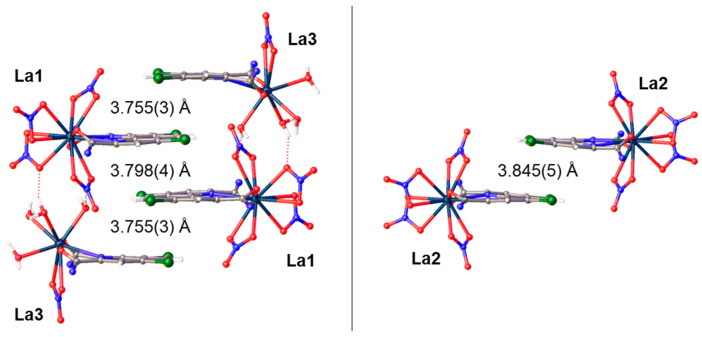
Stacking in the crystal packing of complex La(NO_3_)_3_ with **L**. The central fragments of the complexes are shown for clarity. The centroid–centroid distances are given.

The crystal packing of complex La(NO_3_)_3_ with **L** is defined by a network of hydrogen bonds (D-A distances in the range of 2.537(9)−2.801(7) Å) involving the coordinated water molecules, nitrate oxygen atoms, and free solvent molecules (Figure 10). Moreover, different types of π···π interactions between the phenanthroline fragments stabilize the structure. The complexes La1 and La3 are stacked into centrosymmetric tetramers with centroid–centroid distances of 3.798(4) and 3.755(3) Å. The complex La2 and its symmetric equivalent form dimeric stacks with the distance between plane centroids 3.845(5) Å (Figure 11).

**Figure 12:**
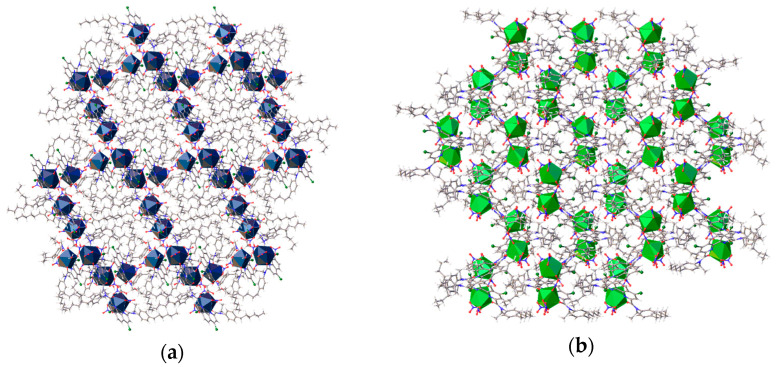
Fragment of the crystal packing of complex of La(NO_3_)_3_ with L. View along a axis (**a**). View along b axis (**b**). The metal atoms are shown as polyhedra.

Hydrogen bonding and stacking lead to the arrangement of a framework consisting predominantly of metal–oxygen cores. The hexyl chains adopting different conformations are combined into distinct hydrophobic areas (Figure 12a), whereas the crystal packings of the neodymium and lutetium complexes are formed only by weak non-directional interaction and do not contain clearly distinguished regions (Figure 12b).

So, the tendency of lanthanum to have higher coordination numbers and the possibilities for hydrogen bonding result in the formation of a completely different packing in the lanthanum complex than that of the lutetium or neodymium complex with the same ligand. However, despite the difference in the structure of the lanthanum complex, this difference did not affect the value of its solubility.

## 3. Materials and Methods

### 3.1. Materials

N,N’-diethyl-N,N’-di(para-hexyl-phenyl)-diamide of 1,10-phenanthroline-4,7-dichloro-2,9-dicarboxylic acid (L) was prepared, purified, and characterized according to the published method [39]. *m*-nitrobenzotrifluoride (F-3) (OJSC “PIM-INVEST”), acetonitrile (ACROS, HPLC grade), chloroform, 1,2-dichloroethane, nitrobenzene, toluene, octanol-1, butanol-1, dodecanol-1 (ACROS) C_4_mimNTf_2_ (Merck, Darmstadt, Germany, >99%); crystalline hydrates of lanthanide nitrates (Sigma-Aldrich, Waltham, MA, USA, 99.9%); and radionuclides ^241^Am, ^152^Eu (Company “Isotope”, Tokyo, Japan) were used in this work. Aqueous solutions were prepared using deionized water (MilliPore Simplicity, Merck, Darmstadt, Germany) and concentrated nitric acid (analytical grade, Chimmed Group, Moscow, Russia) were employed.

### 3.2. Methods

#### 3.2.1. Synthesis and Analysis of Complex Compounds

Complex compounds were synthesized by adding a solution of lanthanide nitrate (0.5 mmol) in dry acetonitrile to a solution of the ligand **L** (0.5 mmol) in chloroform (V = 1 mL) under stirring. Subsequently, the reaction mixture was concentrated in vacuo (at ~20 Torr), treated with 2–3 mL of diethyl ether, and the resulting precipitate of the complex was filtered off and washed with a fresh portion of ether. The obtained complex was air-dried to a constant weight. To confirm the complex nature of the obtained compounds, IR spectroscopy techniques were employed using a Nicolet iS5 FT-IR spectrometer (Thermo Scientific, Waltham, MA, USA) equipped with an attenuated total reflectance (ATR) featuring a diamond optical element (resolution 4 cm^−1^, number of scans 32). Additionally, the melting points of the resulting compounds were determined using a melting point apparatus (Büchi melting point apparatus Model B-545).

NMR spectra were recorded using standard 5 mm sample tubes on Agilent 400-MR spectrometer (Agilent, Santa Clara, CA, USA) with operating frequencies of 400.1 MHz (1H). NMR (Figures S1–S4) and IR (Figures S5–S9) spectra and characterization (Table S5) for all complexes and pure **L** are shown in Supplementary Materials. The IR spectra of all the obtained complexes show a shift of the C=O band by about 40 cm^−1^ compared to the ligand **L** (Table S6), which indicates the formation of metal bonds with the coordination centers of diamides.

#### 3.2.2. Solubility of **L** and Complex Compounds

The solubility of **L** and their complexes with lanthanides in different diluents was determined using the following procedure: a suspended sample of the ligand/complex with a known mass (5 mg) was placed in an Eppendorf tube, and the organic solvent was added incrementally. After each addition of a small volume of solvent, the sample was placed on a vortex shaker for 10 min. The process was repeated until the substance visually dissolved. The volume of the resulting solution was then measured.

HRMS ESI—mass spectra were recorded on the MicroTof Bruker Daltonics and Orbitrap Elite instruments (Bruker, Billerica, MA, USA). The LC system consisted of two LC-20AD pumps (Shimadzu, Tokyo, Japan), and an autosampler was coupled on-line with an LCMS-IT-TOF mass spectrometer equipped with an electrospray ionization source (Shimadzu, Tokyo, Japan). The analysis was carried out without a chromatographic column. Time analysis was 1 min. The mobile phase consisted of HPLC-grade acetonitrile at a flow rate of 0.3 mL/min. Mass spectra were obtained in two *m*/*z* ranges from 200 to 800 and from 800 to 1300 Da. The following parameters were used during analysis: CDL and heat block temperature was 548 K; nebulizing gas flow 1.5 mL/min; positive ion mode, interface voltage 4.5 kV; ion accumulation time 30 ms.

#### 3.2.3. Extraction Experiments

Extraction experiments were performed in 1.5 mL polypropylene Eppendorf tubes. The organic phase (0.5 mL) and the aqueous phase (0.5 mL) were intensively mixed with a Vortex shaker in a thermostat (T = 298 ± 1 K). After that, the samples were centrifuged (5 min, 9000 rpm), and aliquots of 0.4 mL were taken for further analysis. For back-extraction experiment, organic phase was contacted with equal volume of 0.05 mol/L HNO_3_. After centrifugation, these aliquots were analyzed.

For saturation of organic phases, aqueous phases containing 0.1 mol/L and 0.5 mol/L stable Eu^3+^ with addition of ^152^Eu^3+^ were used. The content of ^241^Am (E_γ_ = 59.5 keV) and ^152^Eu (E_γ_ = 121.8 keV) was determined by γ-spectrometry (ORTEC DSPec50 radiometric complex with a coaxial gamma detector, Ametek, Berwyn, PA, USA). The radionuclide contents in the initial aqueous phase were ∼1500 and ∼2500 Bq/mL for ^241^Am and ^152^Eu, respectively.

Quantitative determination of lanthanides in the initial aqueous phase (C_0_) and in the aqueous phase after extraction (C) was carried out using ICP-MS (Analytic Jena Plasma Quant MS Elite, Analytik Jena, Jena, Germany). The concentration of each lanthanide was 0.1 mmol/L. The distribution ratio D is equal to the ratio of the element concentrations in the organic and aqueous phases, respectively. The separation factor SF(M1/M2) is equal to the distribution ratio of two different elements.

#### 3.2.4. UV-Visible Titration of **L** and f-Elements Complexes

The spectra were recorded at 298.0 ± 0.1 K in the wavelength range of 200–500 nm using a spectrophotometer (Shimadzu UV 1900i, Shimadzu, Tokyo, Japan) equipped with a thermostatic attachment (Shimadzu TCC-100, Shimadzu, Tokyo, Japan). Quartz cuvettes with an optical path length of 10 mm were utilized. Working solutions of the metal (Eu^3+^) and ligand **L** were prepared by dissolving the suspension in a molecular organic solvent, each with concentrations of 10^−3^ mol/L and 10^−5^ mol/L, respectively. For the titration, 2 mL of the working solution of the extractant was used, and 2 µL of the metal cation working solution was added incrementally until the change in the appearance of the absorption spectrum ceased. The titration was repeated three times to confirm the reproducibility of the results. The data obtained during spectrophotometric titration were processed using the HypSpec2014 program [38].

#### 3.2.5. XRD Method

The complex substances were prepared by mixing the solutions of components (the ratio of components corresponded to stoichiometry in expected complexes) or dissolving synthesized complexes in dried acetonitrile, chloroform, toluene, or their mixture. The crystal structures of these substances were prepared via slow isothermal evaporation. Crystallographic data for complex La(NO_3_)_3_ with **L** were collected on a Bruker D8 Venture diffractometer (Bruker, Billerica, MA, USA) using graphite monochromatized CuKα radiation (λ = 1.54178 Å) in ω-scan mode at T = 100 K. Crystallographic data for NdL(NO_3_)_3_ and LuL(NO_3_)_3_ were collected on a Bruker D8 Venture and a Bruker Smart Photon II diffractometer using graphite monochromatized MoKα radiation (λ = 0.71073 Å) in ω-scan mode at T = 150 K.

Cell refinement and data reduction for all structures were conducted using the software SAINT (V8.38A, Bruker, Yokohama City, Japan). Absorption correction based on measurements of equivalent reflections was applied (SADABS-2016/2, Bruker 2016/2). The structures were solved by direct methods (SHELXT 2018/2) [40] and refined by full-matrix least-squares on F2 (SHELXL 2018/3) [41] with anisotropic displacement parameters for all non-hydrogen atoms, except some minor components of disordered groups and partially occupied solvent molecules in LuL(NO_3_)_3_ and complex La(NO_3_)_3_ with **L**, which were refined with isotropic displacement parameters. Hydrogen atoms were placed in calculated positions and refined using a riding model. NdL(NO_3_)_3_ and LuL(NO_3_)_3_ were treated as inversion twins with the second domain fractions of 0.479(8) and 0.450(6), respectively. The component ratios in all disordered groups were first refined and then fixed in the final refinement. For crystallographic details, see Tables S7–S9.

## 4. Conclusions

In this work, the influence of solvent nature on the separation parameters of Am(III) and Ln(III) in extraction systems based on N,O-donor ligand **L** was investigated. The distribution ratios of Am(III) and Ln(III) in various molecular solvents: chloroform, 1,2-dichloroethane, toluene, nitrobenzene, octanol-1, dodecanol-1, *m*-nitrobenzotrifluoride (F-3), and ionic liquid C_4_mimNTf_2_ were determined. The resulting values of D(Am) increase from 1.4 to 537 and D(Eu) from 0.08 to 13.4 when using diluents with larger values of dielectric constant. However, the dependence is not linear. The values of SF(Am/Eu) for all solvents are in the region of 17–40 and do not correlate with dielectric constant values. The values of binding constants obtained in different solvents do not contradict these data.

The mechanism of complexation in each solvent was investigated. The solvation numbers for Eu(III) and Am(III) with **L** in each solvent were determined, and the spectrophotometric titration of Eu(III) with **L** was carried out. It is shown that depending on the organic solvent used, the stoichiometry of complexes of *f*-elements with ligand **L** is different. Equilibrium between ML(NO_3_)_3_ and ML_2_(NO_3_)_3_ complexes can exist in the organic phase; the ML(NO_3_)_3_ complex predominates in most cases. However, in the case of toluene and chloroform, the clear predominance of the 1:2 complex was observed for Am(III). In the case of ionic liquid, americium and europium form ML_2_(NO_3_)_3_ complexes preferably.

It is worth noting that the form of the complex also depends on the *f*-element since, in the case of La(III), we observed a complex compound consisting of cationic and anionic parts, unlike complexes of other *f*-elements. Nevertheless, the dissolution of these compounds in various solvents was similar and did not depend on the metal.

An important parameter is the phase stability of the extraction system—a third phase in the case of excess of metal formed in systems with aliphatic alcohols and ionic liquid, unlike all other solvents (aromatic and halogen-containing).

Choosing the diluent is an important step in separation technology modeling due to the influence of physical and chemical properties on the extraction parameters of **L** and the safety of the operator. Table S9 presents a sum of all that was discussed in this research article. The compromise variants for diamides of 1,10-phenanthroline in this list are nitrobenzene and F-3. These are highly polar aromatic solvents, the disadvantage of which is high transfer to the aqueous phase during extraction and fluoride anion leaching in the case of F-3. Another disadvantage is the toxicity of these compounds. Of course, when it comes to the treatment of high-level waste, the danger of toxicity is a secondary consideration. Nevertheless, it is important to reduce the toxicity of the solvent. Searching for the “ideal” diluent for diamides of 1,10-phenanthroline has not ended.

## Figures and Tables

**Table 1:** Solvation numbers of Am(III) and Eu(III) for **L** in different diluents. Aqueous phase: 3 mol/L HNO_3_. Organic phase: 6.25 mmol/L—0.1 mol/L of **L** (* for ionic liquid C(L): 1.5 mmol/L—0.025 mol/L), number of points = 5, R^2^ = 0.99, t = 15 min. (for ionic liquid t = 1 h), T = 298 ± 1 K (*p* = 0.95; *n* = 3; SD < 10%).

Diluent	Dielectric Constant (ε)	SN (Am)	SN (Eu)
Toluene	2.3	2.0	1.4
Chloroform	4.8	2.0	1.2
Dodecanol-1	6.5	1.5	1.1
Octanol-1	10.2	1.3	1.0
1,2-dichloroethane	10.4	1.5	1.2
C_4_mimNTf_2_ *	14.0	2.0	2.0
F-3	22.3	1.3	1.1
Nitrobenzene	35	1.6	1.3

**Table 2:** Stability constants of ML_n_ (M = Eu^3+^, *n* = 1, 2) complexes in different solvents.

Diluent	Dielectric Constant (ε)	Stability Constant, logβ ML	Stability Constant, logβ ML_2_
Octanol-1	10.2	4.66 ± 0.04	9.83 ± 0.04
Butanol-1	17.8	5.86 ± 0.02	11.08 ± 0.05
Acetonitrile	37.5	7.88 ± 0.08	13.70 ± 0.16
Octanol-1	10.2	4.66 ± 0.04	9.83 ± 0.04

## Data Availability

Data are contained within the article.

## Supplementary Materials

The following supporting information can be downloaded at: https://www.mdpi.com/article/10.3390/molecules29153548/s1, properties of diluents, extraction, NMR, IR, XRD, HRMS ESI data.
