# Supplementary material for: Influence of Diluent on Extraction Parameters of Systems for Separation Am(III) and Ln(III) Based on 1,10-Phenanthroline-2,9-Diamide
Source: Molecules. 2024 Jul 28;29(15):3548. doi: 10.3390/molecules29153548 (PMC11313845; doi:10.3390/molecules29153548)
Supplement: Supplementary file 1 [file molecules-29-03548-s001.zip › molecules-3111066-supplementary.pdf]

1. Properties of diluents

Table S1. Properties of diluents (T=298 K, P=0.1 MPa) and approximate solubility of L in the solvents [8,30,31,37–41].

Diluent	Dielectric constant (ε)	Density, g/mL	Viscosity (η), mPa × s	Molarity, mol/L	Polarizability (π*)*	C(L) in saturation solution, mol/L
Toluene	2.3	0.87	0.59	9.41	0.51	0.68
Chloroform	4.8	1.49	0.57	12.48	0.40	1.18
Dodecanol-1	6.5	0.83	16.62	4.46	0.45	0.14
Octanol-1	10.2	0.82	7.29	6.38	0.73	0.15
1,2-dichloroethane	10.4	1.25	0.83	12.63	0.58	1.05
$C_4mimNTf_2$	14.0	1.43	51.7	3.41	0.99	0.03
F-3	22.3	1.44	2.35	7.51	–	0.30
Nitrobenzene	35	1.20	2.03	9.75	0.86	0.60

* Kamlet-Taft solvent parameters

2. Extraction data

Table S2. D(Am), D(Eu) and SF(Am/Eu) in different diluents. Aqueous phase: 3 mol/L HNO_3. Organic phase: 0.025 mol/L of **L**. t = 15 min., T = 298±1 K (P=0.95; n=3; SD < 10 %).

Diluent	Dielectric constant (ε)	Extraction			Back-extraction		
		D(Am)	D(Eu)	SF(Am/Eu)	D(Am)	D(Eu)	SF(Am/Eu)
Toluene	2.3	3.2 ± 0.2	0.11 ± 0.01	30	0.023 ± 0.002	0.060 ± 0.002	0.4
Chloroform	4.8	1.4 ± 0.1	0.08 ± 0.01	17	0.015 ± 0.001	0.027 ± 0.002	0.6
Dodecanol-1	6.5	10.3 ± 0.4	0.29 ± 0.02	36	8.81 ± 0.2	0.54 ± 0.03	16.4
Octanol-1	10.2	6.7 ± 0.3	0.25 ± 0.02	26	0.072 ± 0.004	0.018 ± 0.002	4.0
1,2-dichloroethane	10.4	12.4 ± 0.5	0.48 ± 0.03	26	0.029 ± 0.003	0.006 ± 0.001	4.6
$C_4mimNTf_2$	14.0	537 ± 20	13.4 ± 0.4	40	1310 ± 30	457 ± 9	2.9
F-3	22.3	28.0 ± 0.9	0.90 ± 0.06	31	0.075 ± 0.005	0.006 ± 0.001	12.5
Nitrobenzene	35	57 ± 2	2.4 ± 0.1	23	0.28 ± 0.02	0.008 ± 0.001	32.5

Table S3. Values of D(Ln) in different diluents. Aqueous phase: 3 mol/L HNO_3. Organic phase: 0.025 mol/L of **L**. t = 15 minutes (for ionic liquid t = 1 hour), T = 298±1 K (P=0.95; n=10; number of scans = 10 SD < 2 %).

**Diluent**	**Toluene**	**Chloroform**	**Dodecanol-1**	**Octanol-1**	**1.2-dichloroethane**	**$C_4mimNTf_2$***	**F-3**	**Nitrobenzene**
**ε**	2.3	4.8	6.5	10.2	10.4	14	22.3	35
**La**	24.74	14.63	11.03	13.23	60.66	1325.44	155.28	118.16
**Ce**	10.28	6.59	8.12	8.27	25.01	1180.10	68.38	154.96
**Pr**	3.29	2.29	3.87	4.11	10.76	490.22	31.49	23.77
**Nd**	0.91	0.67	1.65	1.60	3.42	184.11	10.85	11.05
**Sm**	0.11	0.16	0.26	0.24	0.87	78.79	2.48	4.99
**Eu**	0.05	0.09	0.14	0.11	0.56	53.99	1.62	3.24
**Gd**	0.07	0.08	0.11	0.07	0.32	38.47	0.94	1.67
**Tb**	0.03	0.04	0.07	0.05	0.26	36.96	0.81	1.51
**Dy**	0.03	0.04	0.06	0.04	0.20	33.68	0.63	1.13
**Ho**	0.03	0.02	0.09	0.07	0.14	31.28	0.49	0.74
**Er**	0.02	0.01	0.07	0.05	0.09	30.15	0.38	0.49
**Tm**	0.02	0.00	0.05	0.04	0.05	29.72	0.26	0.31
**Yb**	0.02	0.01	0.04	0.02	0.03	28.93	0.22	0.21
**Lu**	0.03	0.02	0.03	0.03	0.04	25.30	0.19	0.19

Solvation numbers

The extraction equilibrium can be described by the following equation:

$$M^{3+}_{(aq.)} + 3NO^{-}_{3(aq.)} + nL_{(org.)} \leftrightarrow M(NO_3)_3(L)_{n(org.)} \quad (S1)$$

, where M^{3+} – ion of metal, L – ligand, n – quantity of ligand molecules per metal ion (solvation number).

Extraction equilibrium constant (K_{ex}):

$$K_{ex} = \frac{[M(NO_3)_3(L)_{n(org.)}]}{[M^{3+}_{(aq.)}][NO^{-}_{3(aq.)}]^3[L_{(org.)}]^n} = \frac{D}{[NO^{-}_{3(aq.)}]^3[L_{(org.)}]^n} \quad (S2)$$

Logarithmizing the extraction equilibrium constant, we obtain the following equation:

$$lgD = nlg[L_{(org.)}] + lgK_{ex} + 3\,lg[NO^{-}_{3(aq.)}] = nlg[L_{(org.)}] + Const \quad (S3)$$

The concentration of nitric acid in the equilibrium aqueous phase was constant at 3 mol/L. Hence, Const = $lgK_{ex} + 3\,lg[NO^{-}_{3(aq.)}]$.

Table S4. Equations lgD(Me)=n×lgC(L)+const for **L** in different diluents. Aqueous phase: 3 mol/L HNO_3. Organic phase: 6.25 mmol/L - 0.1 mol/L of L (*for ionic liquid C(**L**): 1.5 mmol/L – 0.025 mol/L), number of points = 5, R^2 = 0.99. t = 15 min (* for ionic liquid t = 1 hour), T = 298 ± 1 K (for every measurement: P=0.95; n=3; SD < 10 %).

**Diluent**	**Am(III)**			**Eu(III)**		
	**n**	**const**	**R^2**	**n**	**const**	**R^2**
Toluene	1.91	3.57	0.971	1.40	1.27	0.989
Chloroform	2.03	3.39	0.975	1.17	0.77	0.931
Dodecanol-1	1.46	3.35	0.971	1.10	1.22	0.983
Octanol-1	1.32	2.94	0.961	1.04	1.07	0.967
1,2-dichloroethane	1.47	3.45	0.948	1.17	1.56	0.978
$C_4mimNTf_2$*	2.01	5.95	0.998	2.00	4.33	0.999
F-3	1.30	3.53	0.952	1.07	1.67	0.985
Nitrobenzene	1.59	4.30	0.994	1.30	2.47	0.991

3. NMR spectra of synthesized compounds

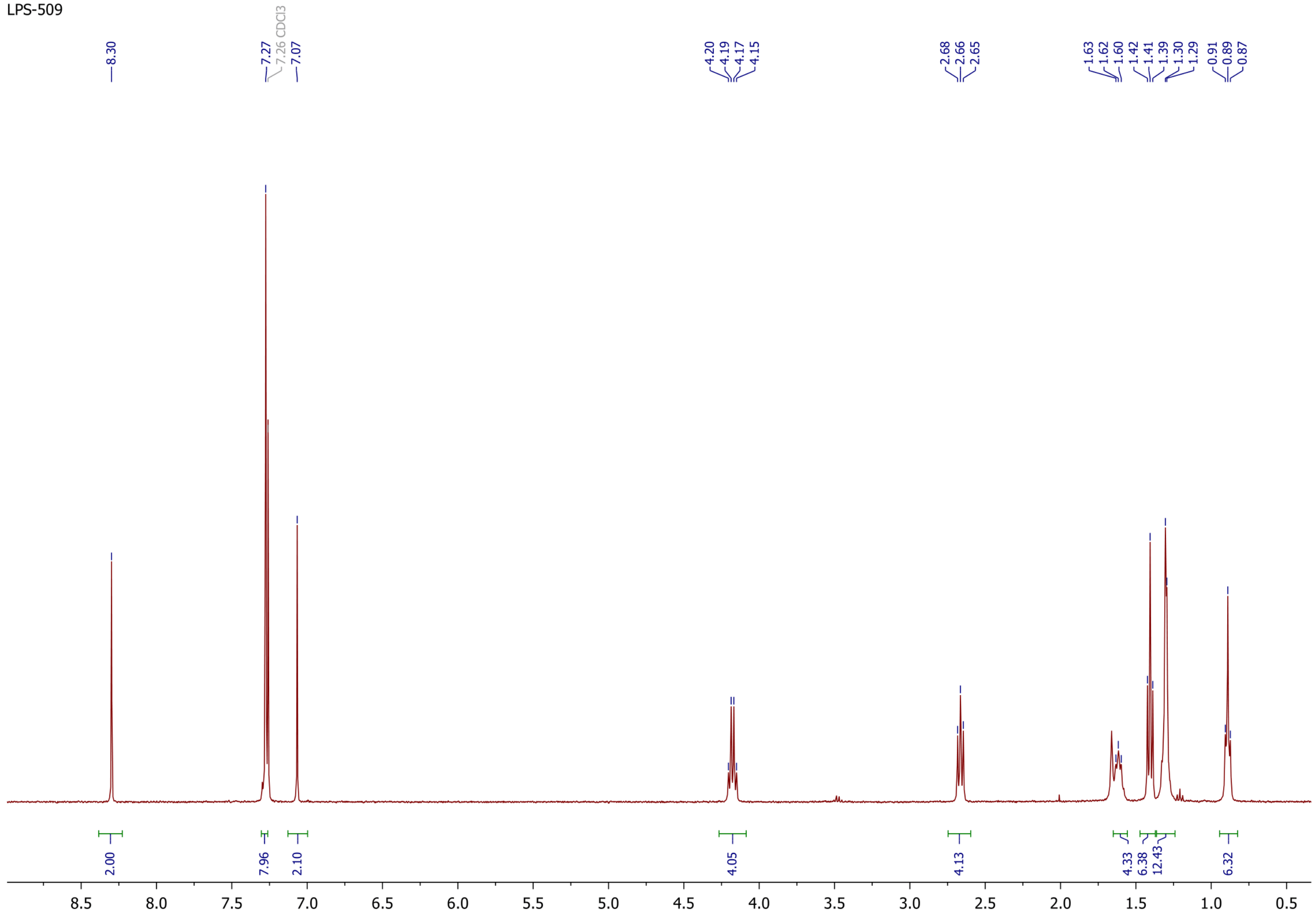

Figure S1. ^{1}H NMR spectra of complex $LaL(NO_3)_3$ in $CDCl_3$ at 298 K

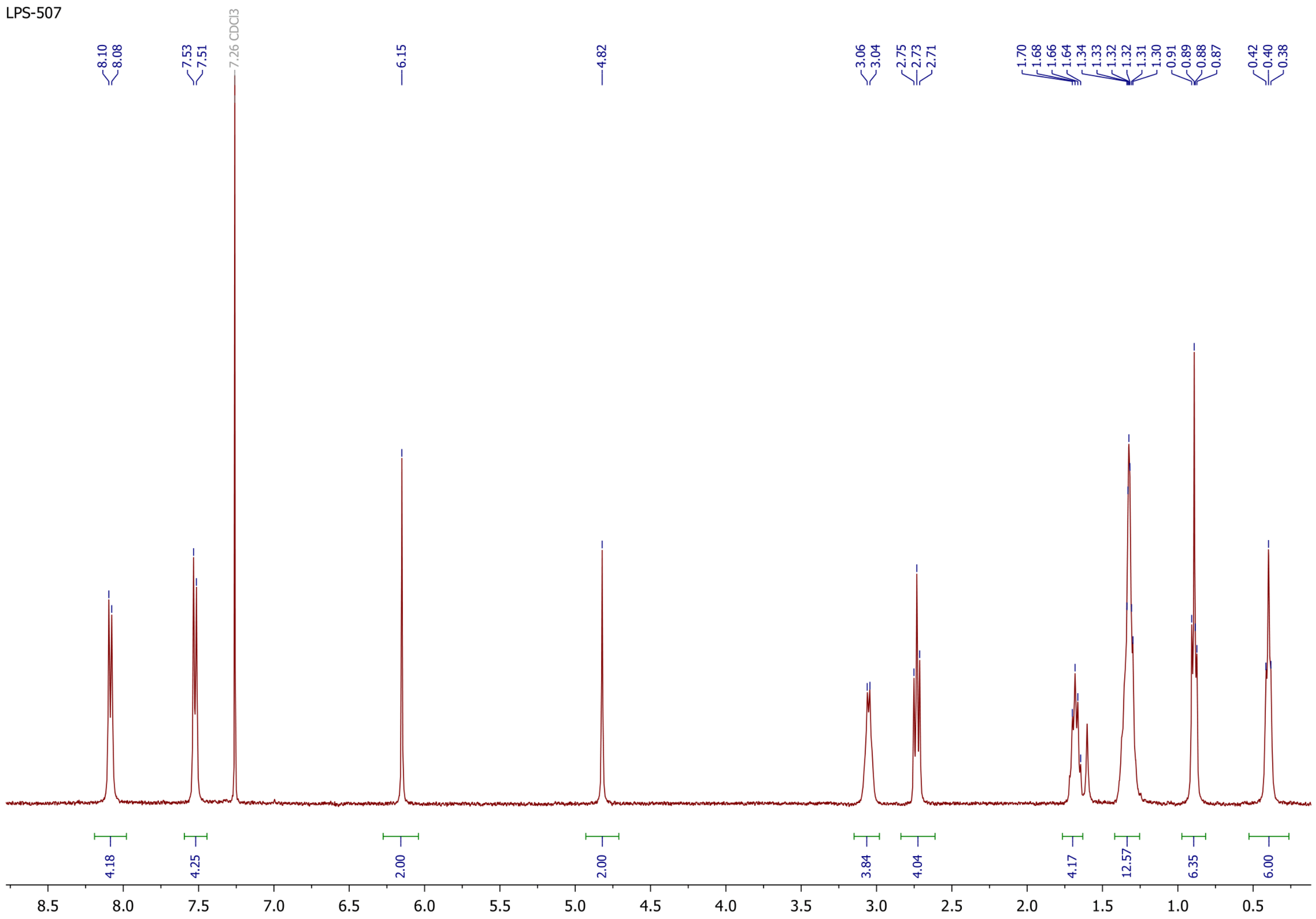

Figure S2. ^{1}H NMR spectra of complex $EuL(NO_3)_3$ in $CDCl_3$ at 298 K

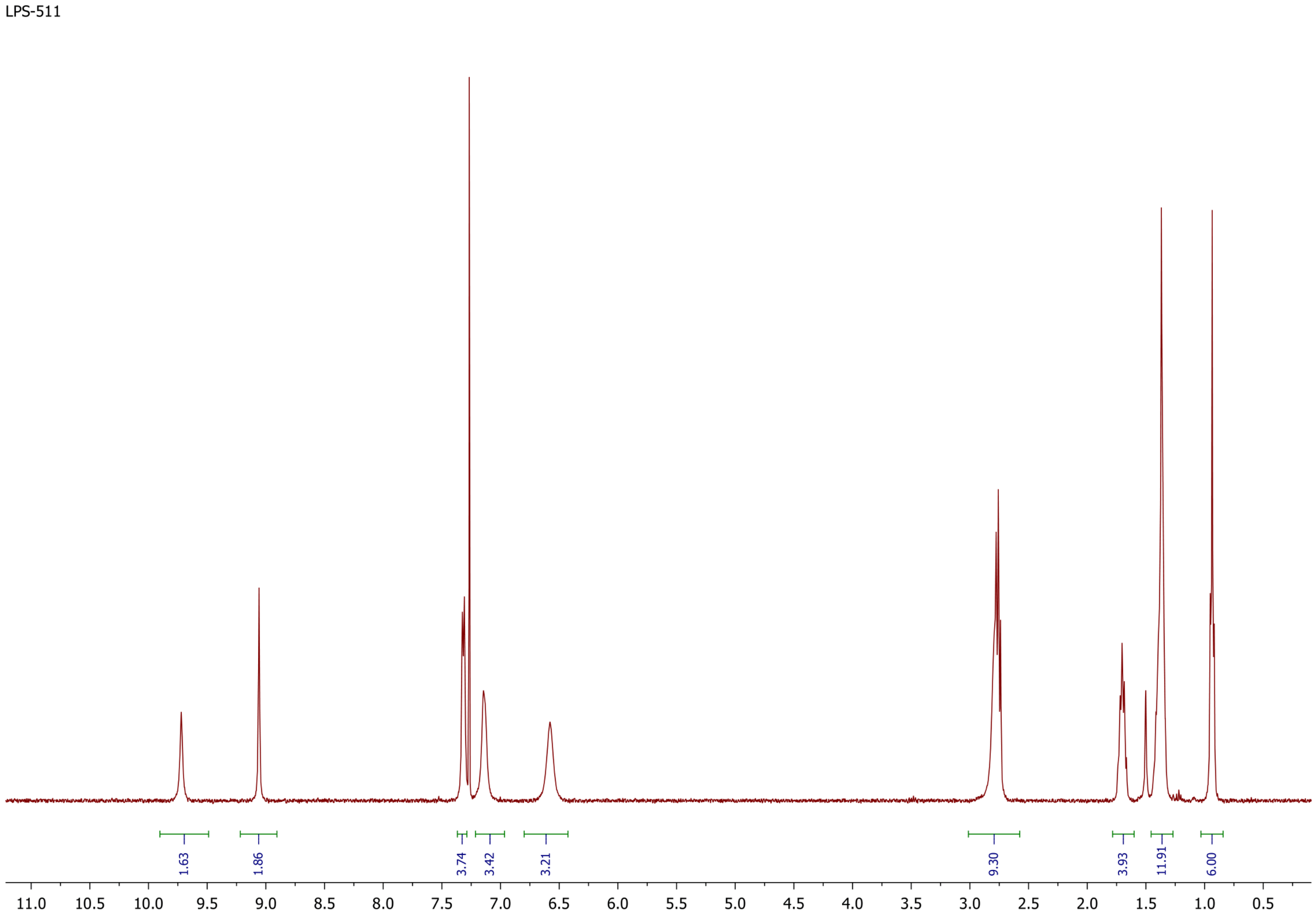

Figure S3. ^1H NMR spectra of complex $NdL(NO_3)_3$ in $CDCl_3$ at 298 K

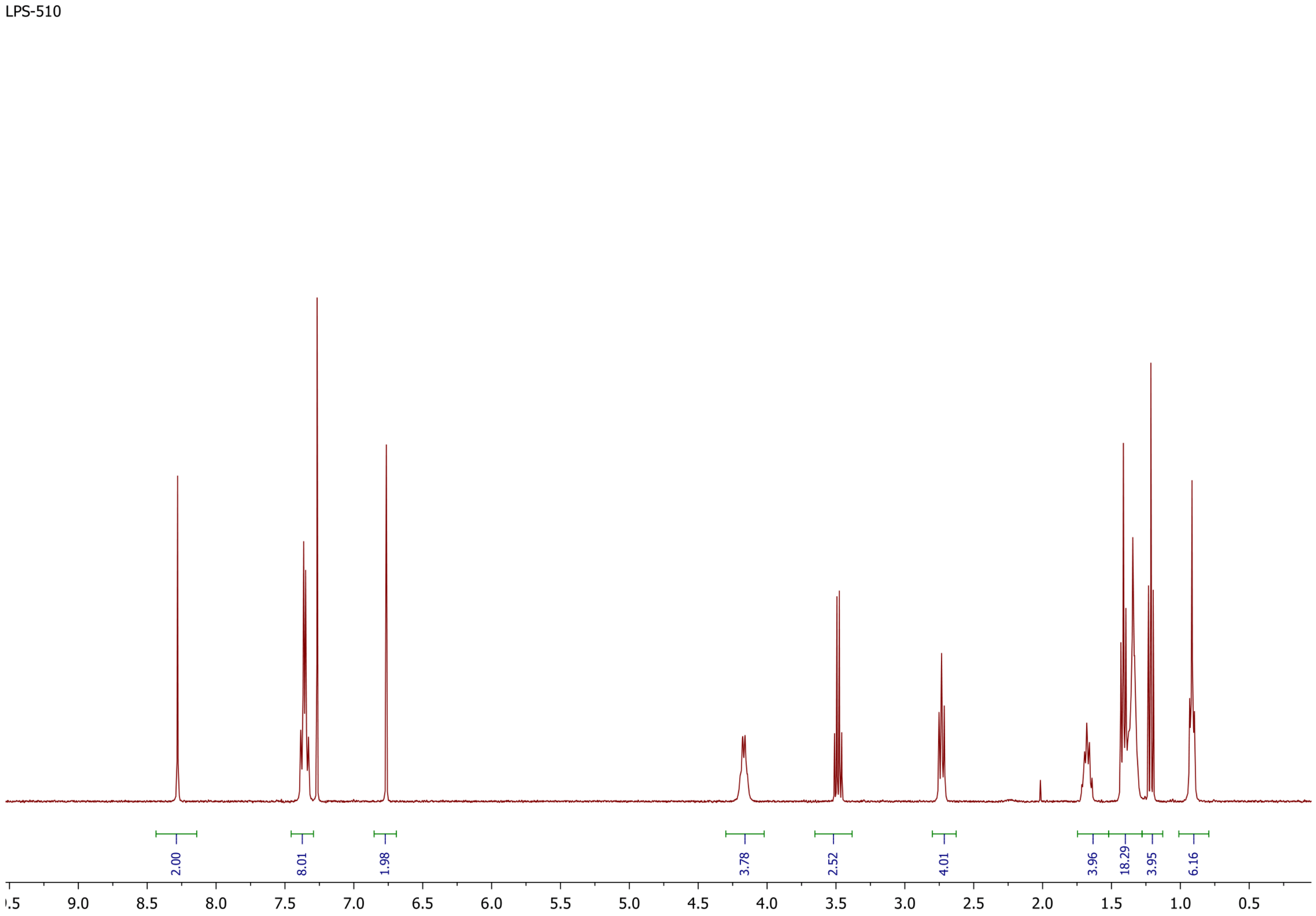

Figure S4. ^{1}H NMR spectra of complex $LuL(NO_3)_3$ in $CDCl_3$ at 298 K

Table S5. Characterization of complex substances

Complex	Description	$T_{decomp.}$, K	^{1}H NMR (400 MHz, Chloroform-*d*)
$LaL(NO_3)_3$	pale yellow powder	473	δ 8.30 (s, 2H), 7.27 (s, 8H), 7.07 (s, 2H), 4.18 (q, $^3J_{HH}$ = 7.1 Hz, 4H), 2.66 (t, $^3J_{HH}$ = 7.5 Hz, 4H), 1.67 – 1.57 (m, 4H), 1.41 (t, $^3J_{HH}$ = 7.1 Hz, 6H), 1.33 – 1.26 (m, 12H), 0.92 – 0.85 (t, $^3J_{HH}$ = 6.6 Hz 6H).
$EuL(NO_3)_3$	light yellow powder	473	δ 8.09 (d, $^3J_{HH}$ = 7.7 Hz, 4H), 7.52 (d, $^3J_{HH}$ = 7.4 Hz, 4H), 6.15 (s, 2H), 4.82 (s, 2H), 3.05 (q, $^3J_{HH}$ = 6.4, 4H), 2.73 (t, $^3J_{HH}$ = 7.6 Hz, 4H), 1.68 (p, J = 7.4 Hz, 4H), 1.32 (m, 12H), 0.89 (t, $^3J_{HH}$ = 6.4 Hz, 6H), 0.40 (t, $^3J_{HH}$ = 6.6 Hz, 6H)
$NdL(NO_3)_3$	pale green powder	470	δ 9.71 (s, 2H), 9.05 (s, 2H), 7.31 (d, $^3J_{HH}$ = 7.0 Hz, 4H), 7.13 (br. s., 4H), 6.57 (br. s, 4H), 2.87 – 2.67 (m, 9H), 1.69 (q, $^3J_{HH}$ = 7.3 Hz, 4H), 1.44 – 1.30 (m, 12H), 0.93 (t, $^3J_{HH}$ = 7.0 Hz, 6H).
$LuL(NO_3)_3$	pale yellow powder	479	δ 8.28 (s, 2H), 7.36 (d, $^3J_{HH}$ = 8.4 Hz, 4H), 7.34 (d, $^3J_{HH}$ = 8.3 Hz, 4H), 6.76 (s, 2H), 4.17 (q, $^3J_{HH}$ = 7.4 Hz, 4H), 3.48 (q, $^3J_{HH}$ = 7.0 Hz, 3H), 2.73 (t, $^3J_{HH}$ = 7.6 Hz, 4H), 1.68 (p, J = 7.5 Hz, 4H), 1.41 (t, J = 7.2 Hz, 6H), 1.38 – 1.31 (m, 12H), 1.21 (t, $^3J_{HH}$ = 7.0 Hz, 4H), 0.92 (t, $^3J_{HH}$ = 7.1 Hz, 6H).

4. IR spectra of synthesized compounds

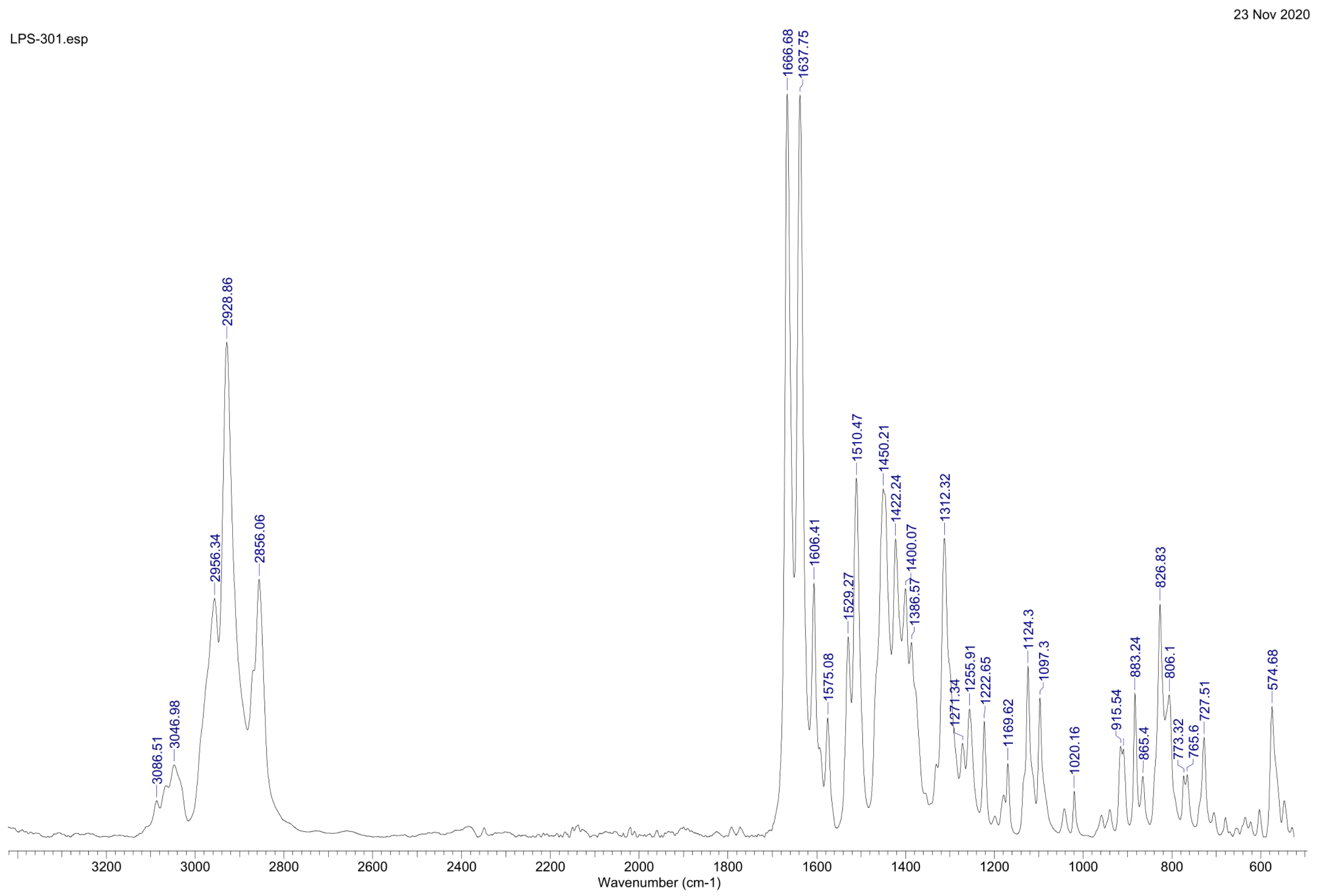

Figure S5. IR spectra of pure L

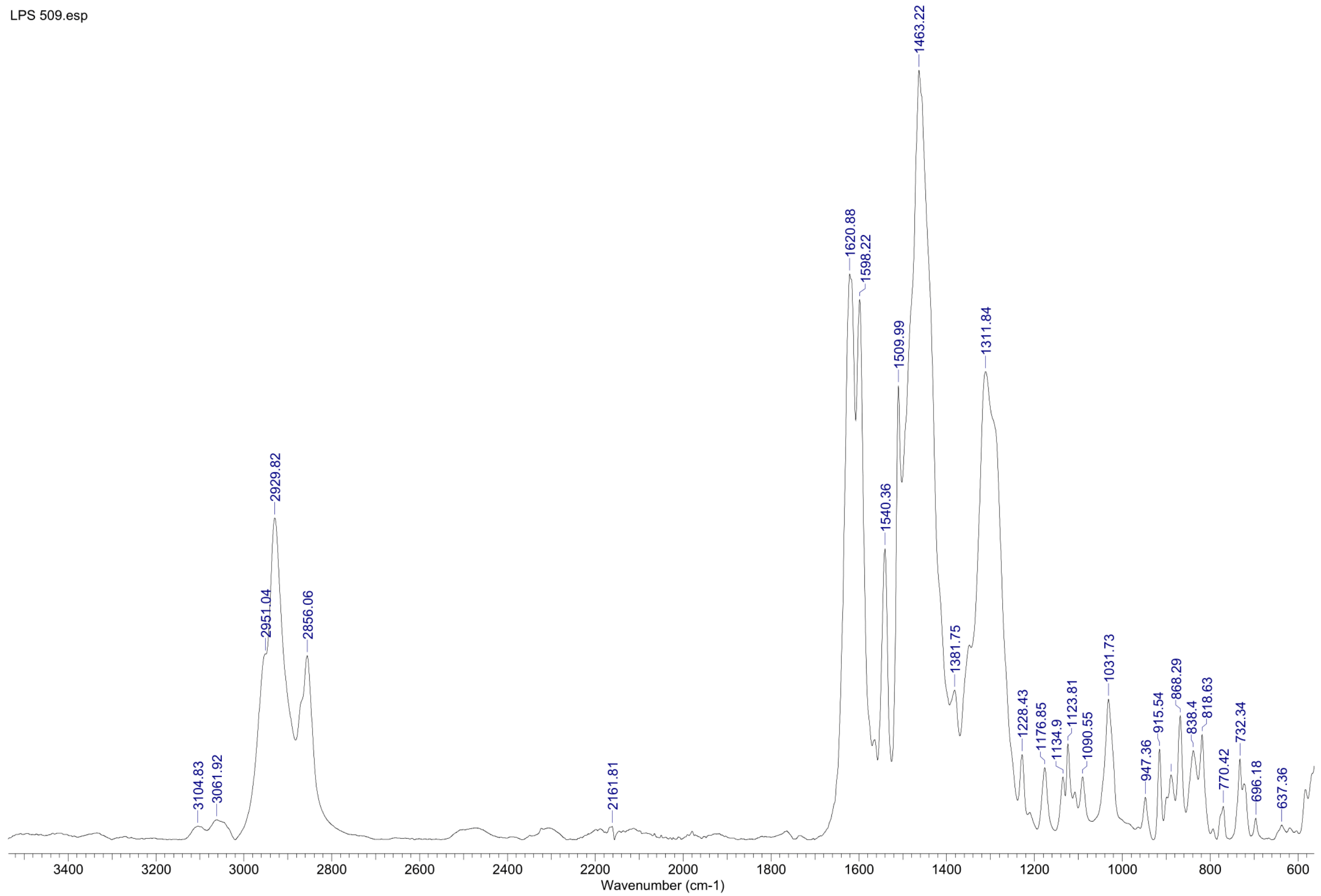

Figure S6. IR spectra of complex $LaL(NO_3)_3$

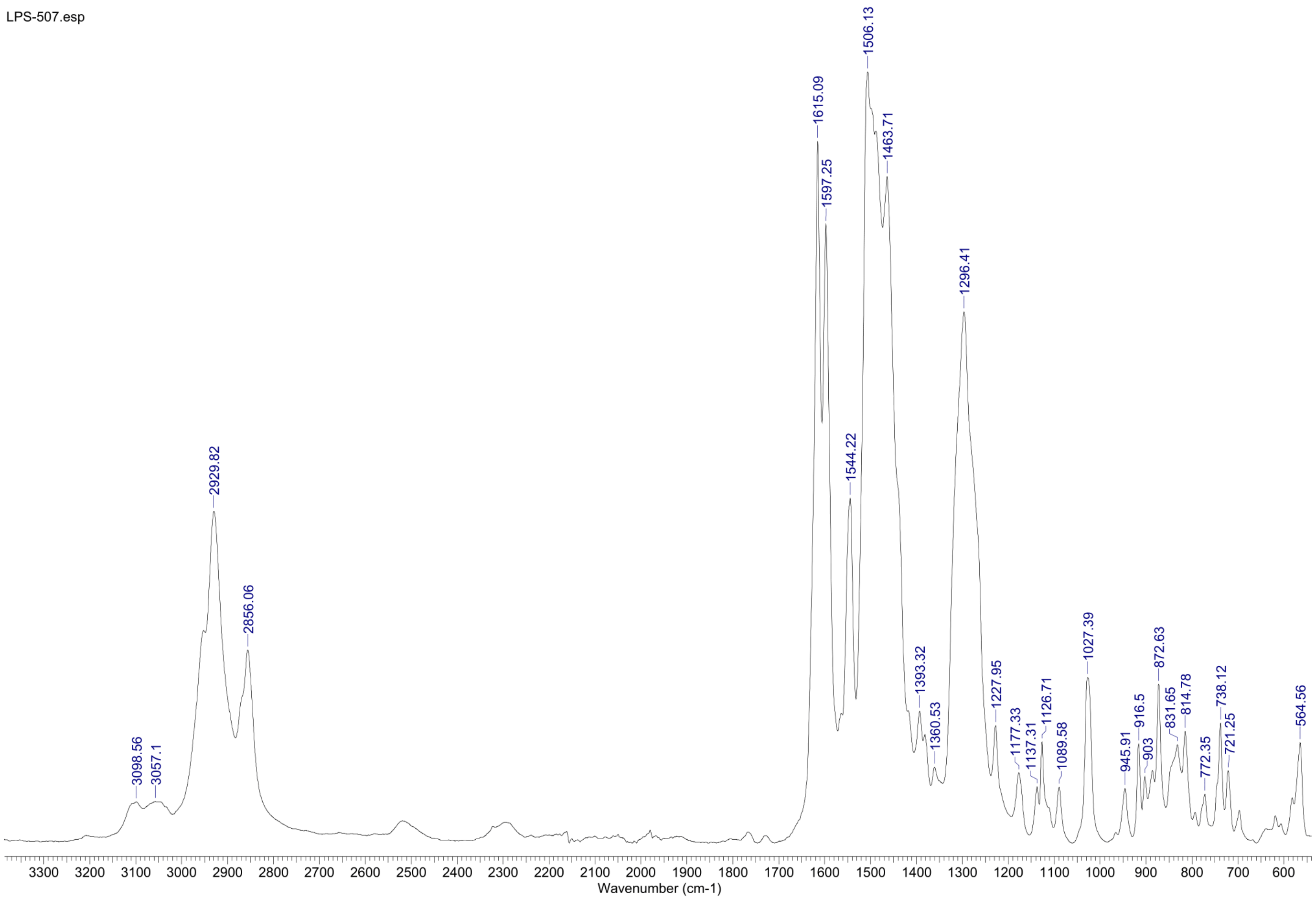

Figure S7. IR spectra of complex $EuL(NO_3)_3$

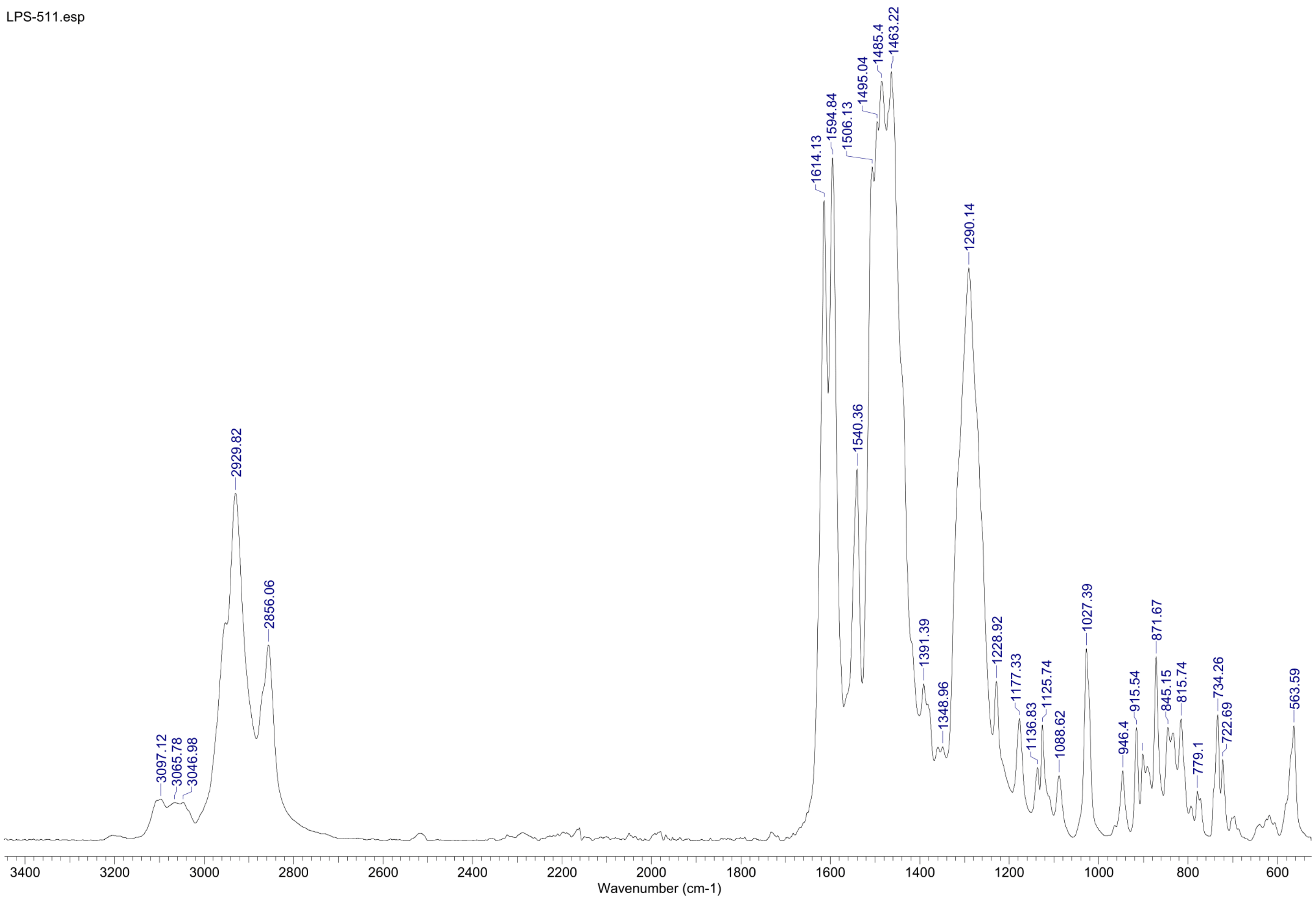

Figure S8. IR spectra of complex $NdL(NO_3)_3$

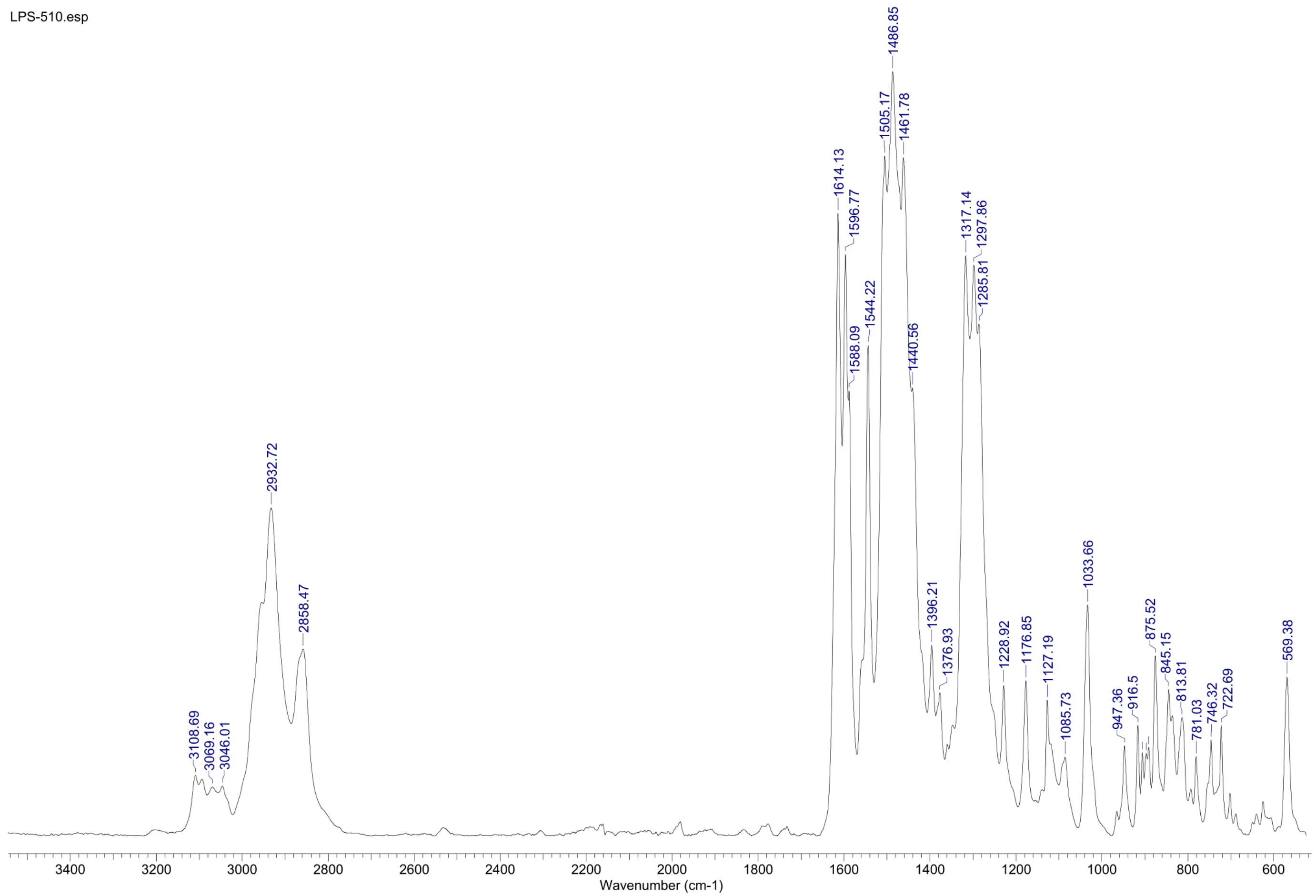

Figure S9. IR spectra of complex $LuL(NO_3)_3$

Table S6. IR lines ($\nu_{C=O}$, cm^{-1}) and $\Delta\nu_{C=O}$ (shift of **L** line after complex formation), cm^{-1} for L and their complexes

	**L**	**LaL(NO$_3$)$_3$**	**NdL(NO$_3$)$_3$**	**EuL(NO$_3$)$_3$**	**LuL(NO$_3$)$_3$**
$\nu_{C=O}$	1637	1599	1595	1597	1597
$\Delta\nu_{C=O}$		38	42	40	40

5. XRD data

Table S7. Crystallographic details for complex $La(NO_3)_3$ with **L**.

**Identification code**	**Complex $La(NO_3)_3$ with L**
**Empirical formula**	$C_{133.5}H_{178.2}Cl_6La_3N_{21}O_{42.6}$
**Formula weight**	3388.19
**Temperature/K**	100
**Crystal system**	triclinic
**Space group**	P-1
**a/Å**	16.0458(5)
**b/Å**	20.7462(7)
**c/Å**	24.5968(8)
**α/°**	71.052(2)
**β/°**	80.188(2)
**γ/°**	82.741(2)
**Volume/Å³**	7608.9(4)
**Z**	2
**ρ_{calc}g/cm³**	1.479
**μ/mm⁻¹**	8.071
**F(000)**	3480.0
**Crystal size/mm³**	0.2 × 0.18 × 0.01
**Radiation**	CuKα (λ = 1.54178)
**2Θ range for data collection/°**	3.834 to 134.998
**Reflections collected**	138284
**Data/restraints/parameters**	27206/24/1896
**Goodness-of-fit on F^2**	1.041
**Final R indexes [I>=2σ (I)]**	R_1 = 0.0557, wR_2 = 0.1425
**Final R indexes [all data]**	R_1 = 0.0787, wR_2 = 0.1609
**Largest diff. peak/hole / e Å⁻³**	2.42/-0.94

Table S8. Crystallographic details for $NdL(NO_3)_3$ and $LuL(NO_3)_3$.

Identification code	$NdL(NO_3)_3$	$LuL(NO_3)_3$
Empirical formula	$C_{42}H_{48}Cl_2N_7NdO_{11}$	$C_{42}H_{48}Cl_2LuN_7O_{11}$
Formula weight	1042.01	1072.74
Temperature/K	150	150
Crystal system	orthorhombic	orthorhombic
Space group	$Pca2_1$	$Pca2_1$
a/Å	25.3185(8)	25.1828(15)
b/Å	20.1609(6)	19.9419(12)
c/Å	17.5178(6)	17.5323(9)
α/°	90	90
β/°	90	90
γ/°	90	90
Volume/Å^3	8941.9(5)	8804.6(9)
Z	8	8
ρ_{calc}g/cm^3	1.548	1.619
μ/mm^{-1}	1.348	2.432
F(000)	4248.0	4336.0
Crystal size/mm^3	0.37 × 0.28 × 0.18	0.21 × 0.13 × 0.12
Radiation	MoKα (λ = 0.71073)	MoKα (λ = 0.71073)
2Θ range for data collection/°	2.828 to 57	3.49 to 61.124
Reflections collected	140894	118405
Data/restraints/parameters	21614/52/1203	26406/43/1157
Goodness-of-fit on F^2	1.025	1.062
Final R indexes [I>=2σ (I)]	R_1 = 0.0343, wR_2 = 0.0640	R_1 = 0.0370, wR_2 = 0.0640
Final R indexes [all data]	R_1 = 0.0482, wR_2 = 0.0685	R_1 = 0.0536, wR_2 = 0.0723
Largest diff. peak/hole / e Å^{-3}	0.91/-0.64	0.90/-0.87
Flack parameter	-0.003(4)	0.000(3)

Table S9. Metal – Oxygen, Metal – Nitrogen distances for complexes

Distance		Å
La1	O1	2.579(4)
La1	O2	2.555(4)
La1	O3	2.691(4)
La1	O4	2.625(4)
La1	O6	2.588(4)
La1	O7	2.806(5)
La1	O9	2.576(4)
La1	O10	2.670(4)
La1	O12	2.700(4)
La1	O13	2.702(4)
La1	N1	2.764(4)
La1	N2	2.785(4)
La2	O15	2.548(4)
La2	O16	2.602(4)
La2	O17	2.696(4)
La2	O18	2.689(4)
La2	O20	2.634(4)
La2	O21	2.732(4)
La2	O23	2.660(4)
La2	O24	2.672(4)
La2	O26	2.636(5)
La2	O27	2.630(4)
La2	N9	2.796(5)
La2	N10	2.766(5)
La3	O29	2.556(4)
La3	O30	2.683(8)
La3	O30A	2.425(18)
La3	O31	2.623(4)
La3	O32	2.669(4)
La3	O34	2.531(4)
La3	O35	2.523(4)
La3	O36	2.527(7)
La3	O37	2.556(6)
La3	N17	2.771(5)

Distance		Å
Nd1	O1	2.429(3)
Nd1	O2	2.413(3)
Nd1	O3	2.556(4)
Nd1	O4	2.544(3)
Nd1	O6	2.565(4)
Nd1	O7	2.538(4)
Nd1	O9	2.555(4)
Nd1	O10	2.552(4)
Nd1	N1	2.586(4)
Nd1	N2	2.622(4)
Nd2	O12	2.464(4)
Nd2	O13	2.428(4)
Nd2	O14	2.548(4)
Nd2	O15	2.511(4)
Nd2	O17	2.551(9)
Nd2	O17A	2.54(2)
Nd2	O18	2.480(11)
Nd2	O18A	2.643(16)
Nd2	O20	2.531(4)
Nd2	O21	2.540(4)
Nd2	N8	2.637(4)
Nd2	N9	2.678(4)

Distance		Å
Lu1	O1	2.321(4)
Lu1	O2	2.313(4)
Lu1	O3	2.444(5)
Lu1	O4	2.423(4)
Lu1	O6	2.516(5)
Lu1	O7	2.399(5)
Lu1	O9	2.432(4)
Lu1	O10	2.455(4)
Lu1	N1	2.457(5)
Lu1	N2	2.496(4)
Lu2	O12	2.332(4)
Lu2	O13	2.306(4)
Lu2	O14	2.390(4)
Lu2	O15	2.419(4)
Lu2	O17	2.251(7)
Lu2	O20	2.369(5)
Lu2	O21	2.418(5)
Lu2	O23	2.48(2)
Lu2	O24	2.48(2)
Lu2	N8	2.472(5)
Lu2	N9	2.502(4)

6. HRMS ESI

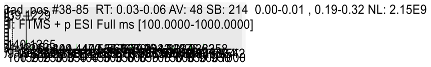

Figure S10. $C_8H_{15}N_2^+$ (**C_4mim$^+$**) spectra (m/z = 139.1229)

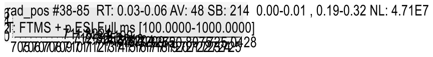

Figure S11. $C_{42}H_{49}O_2N_4{}^{35}Cl_2^+$ (**L**$^+$) spectra (m/z = 711.3221)

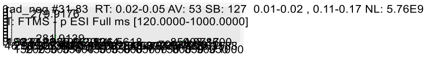

Figure S12. $C_2O_4NF_6{}^{32}S_2^-$ (**NTf$_2^-$**) spectra (m/z = 279.9176)

7. The summary the possibilities of using different diluents in the extraction systems.

Table S9. The pros and cons of different diluents in the extraction systems.

**Diluent**	**Pros**	**Cons**
Toluene	-High solubility of ligand<br>-Phase stability under saturation<br>-Back-extraction	-Volatile
Chloroform	-High solubility of ligand and complexes,<br>-Phase stability under saturation<br>-Back-extraction	-Volatile
1,2-dichloroethane	-High solubility of ligand and complexes,<br>-Phase stability under saturation<br>-Back-extraction	-Volatile
Octanol-1	-Back-extraction<br>-Not toxicity	-Third phase formation<br>-Solubility in water/nitric acid<br>-Specific interaction between **L** and solvent molecules (hydrogen bonds)
Dodecanol-1	-Back-extraction<br>-Not toxicity	-Absence of back-extraction<br>-Third phase formation<br>-Specific interaction between **L** and solvent molecules (hydrogen bonds)<br>-Solubility in water/nitric acid
F-3	-High distribution ratios<br>-Back-extraction	-Price<br>-Toxicity<br>-Solubility in water/nitric acid<br>- Fluoride anion leaching
$C_4mimNTf_2$	-High distribution ratios<br>-Not toxicity	-High viscosity<br>-Third phase formation<br>-Absence of back-extraction<br>-Price<br>-Specific interaction between **L** and solvent molecules<br>-Solubility in water/nitric acid
Nitrobenzene	-High distribution ratios<br>-Back-extraction	-Toxicity<br>-Solubility in water/nitric acid
